# Contemporary Advances (2015–2026) in Extracorporeal Electromagnetic Transduction Therapy and Pulsed Electromagnetic Fields for Musculoskeletal Disorders: A Systematic Review of Dosimetry and Clinical Response

**DOI:** 10.3390/biomedicines14081731

**Published:** 2026-07-31

**Authors:** Ismael Leyva Martínez, Abdi Ramírez Arteaga, Hugo Martínez-Rojano, Víctor Arturo Rocha Herrera, Josué Salvador Aguilar Aja

**Affiliations:** 1 Programa de Doctorado en Investigación en Medicina, Sección de Estudios de Posgrado e Investigación, Escuela Superior de Medicina, Instituto Politécnico Nacional, Prolongación de Carpio y Plan de Ayala s/n, Col. Casco de Santo Tomás, Alcaldía Miguel Hidalgo, Ciudad de México C.P. 11340, Mexico; ismael.leyva@dif.gob.mx; 2 Departamento de Enseñanza e Investigación, Centro Nacional Modelo de Atención, Investigación y Capacitación para la Rehabilitación e Integración Laboral Iztapalapa, Guerra de Reforma y Leyes de Reforma s/n, Col. Leyes de Reforma 3ra Sección, Alcaldía Iztapalapa, Ciudad de México C.P. 09310, Mexico; drarturorocha@gmail.com; 3 Programa de Maestría en Ciencias de la Salud, Sección de Estudios de Posgrado e Investigación, Escuela Superior de Medicina, Instituto Politécnico Nacional, Prolongación de Carpio y Plan de Ayala s/n, Col. Casco de Santo Tomás, Alcaldía Miguel Hidalgo, Ciudad de México C.P. 11340, Mexico; abdiramirez64@gmail.com; 4 Departamento de Valoración y Tratamiento, Centro Nacional Modelo de Atención, Investigación y Capacitación para la Rehabilitación e Integración Laboral Iztapalapa, Guerra de Reforma y Leyes de Reforma s/n, Col. Leyes de Reforma 3ra Sección, Alcaldía Iztapalapa, Ciudad de México C.P. 09310, Mexico; 5 Sección de Estudios de Posgrado e Investigación, Escuela Superior de Medicina, Instituto Politécnico Nacional, Prolongación de Carpio y Plan de Ayala s/n, Col. Casco de Santo Tomás, Alcaldía Miguel Hidalgo, Ciudad de México C.P. 11340, Mexico; 6 Escuela de Terapia Física del Centro de Rehabilitación del DIF Iztapalapa, Centro Nacional Modelo de Atención, Investigación y Capacitación para la Rehabilitación e Integración Laboral Iztapalapa, Guerra de Reforma y Leyes de Reforma s/n, Col. Leyes de Reforma 3ra Sección, Alcaldía Iztapalapa, Ciudad de México C.P. 09310, Mexico; fisioavanzada.ja@gmail.com

**Keywords:** electromagnetic transduction therapy, pulsed electromagnetic fields, musculoskeletal disorders, rehabilitation, electromagnetic dosing, dose–response relationship, physical therapy

## Abstract

**Background and Objective:** Pulsed electromagnetic fields (PEMF) and extracorporeal magnetotransduction therapy (EMTT) have shown promising results in various musculoskeletal disorders; however, substantial heterogeneity in application parameters has hindered the establishment of clear relationships between electromagnetic dosing and clinical response. This systematic review aims to evaluate the safety and effectiveness of PEMF and EMTT therapies for treating chronic musculoskeletal injuries in adults, based on research from 2015 to 2026. By analyzing how specific dosage parameters (such as intensity, frequency, and duration) influence clinical results, the study seeks to establish standardized, evidence-based guidelines for electromagnetic treatment prescriptions. **Methods:** A systematic review was conducted in accordance with PRISMA 2020 guidelines and prospectively registered in PROSPERO (CRD420251059359). A comprehensive search was performed in PubMed/MEDLINE, CENTRAL, and ScienceDirect for the 2015–2025 period. Randomized clinical trials, quasi-experimental studies, and case reports were included. Study selection, data extraction, and risk of bias assessment were performed by two independent reviewers using RoB 2, ROBINS-I, and Joanna Briggs Institute checklists. Results were synthesized narratively following SWiM (Synthesis Without Meta-analysis) recommendations. **Results:** Twenty-eight studies (1060 participants) were included. A dosimetric dichotomy emerged: high-intensity protocols with rapid field variation rates (e.g., EMTT > 60 kT/s) were consistently associated with robust improvements in structural and mechanically driven pathologies. Conversely, low-intensity protocols (e.g., <5 mT) often yielded results indistinguishable from placebo or active controls, particularly in nociplastic pain conditions. Quantitative analysis indicated that in structural pathologies, functional gains were maintained or amplified post-treatment, whereas nociplastic conditions showed potential symptom recurrence. No serious adverse events were reported, confirming a favorable safety profile. **Conclusions:** PEMF and EMTT are promising adjunctive therapies for musculoskeletal disorders, with efficacy appearing sensitive to electromagnetic dosimetry. However, significant methodological heterogeneity and the use of combination therapies limit these findings to exploratory trends. The lack of standardized safety reporting and technical parameters (e.g., waveform, dB/dt) underscores a critical translational gap. Future research must prioritize rigorous, standardized randomized controlled trials to establish definitive, evidence-based therapeutic recommendations.

## 1. Introduction

Musculoskeletal disorders represent a leading global cause of chronic pain and disability, imposing a substantial socioeconomic burden [1,2,3]. Clinically, the primary challenge of these conditions lies not merely in their high incidence, but in the complex pathomechanics driving their progression to chronicity [4]. In highly prevalent disorders such as tendinopathies, pathogenesis is characterized by a detrimental interplay among excessive mechanical stress, persistent localized inflammation, and dysregulated vascularity [5]. This microenvironmental disruption leads to a failed healing response, where normal tissue architecture is replaced by fibrotic changes, persistent nociceptive signaling, and structural degeneration [6,7,8]. Consequently, identifying non-invasive therapeutic strategies capable of directly modulating these aberrant mechanobiological pathways—such as extracorporeal magnetotransduction therapy (EMTT) and pulsed electromagnetic fields (PEMF)—has become a priority in modern musculoskeletal rehabilitation [9].

The progression toward chronicity largely reflects a disruption of the biological mechanisms responsible for tissue repair. Normal recovery of musculoskeletal tissues requires a tightly regulated transition between the inflammatory, proliferative, and remodeling phases, coordinated by a complex interaction among immune cells, cytokines, growth factors, and extracellular matrix components [10,11,12]. Among the primary regulators of this process are macrophages, whose functional plasticity plays an essential role during healing. In the initial stages of injury, a pro-inflammatory (M1) phenotype predominates, responsible for phagocytosing damaged tissue, clearing cellular debris, and releasing mediators such as tumor necrosis factor-alpha (TNF-α), interleukin-1 beta (IL-1β), and interleukin-6 (IL-6) [13]—all of which are indispensable for initiating the reparative response. As healing progresses, these macrophages gradually acquire a pro-resolving (M2) profile, characterized by the secretion of anti-inflammatory cytokines such as interleukin-10 (IL-10), transforming growth factor-beta (TGF-β), and multiple bioactive factors that promote angiogenesis, extracellular matrix synthesis and organization, and functional tissue regeneration [14,15,16]. When this transition is delayed or remains dysregulated, a chronic inflammatory microenvironment persists, sustaining active catabolic pathways and promoting fibrotic deposition [17,18]. The functional relevance of this aborted healing response lies in the direct translation of molecular dysregulation into biomechanical failure; impaired collagen remodeling and compromised structural integrity inevitably manifest clinically as load intolerance, persistent pain, and severe functional impairment [19]. This direct link between a failed M1-M2 macrophage transition and clinical disability provides the primary biological rationale for developing therapeutic strategies aimed at modulating the mechanotransduction pathways that govern tissue repair, rather than focusing exclusively on symptomatic relief [20].

The need to intervene in these biological processes has driven the development of strategies capable of modulating the tissue microenvironment beyond symptomatic relief [21]. Among these, electromagnetic-field-based therapies have garnered increasing interest due to their ability to influence multiple mechanisms related to inflammation, cellular proliferation, angiogenesis, extracellular matrix organization, and tissue regeneration [22,23,24]. Unlike treatments focused exclusively on analgesia, these modalities seek to promote a biological environment more conducive to repair, acting on cellular processes involved in both the resolution of inflammation and the structural restoration of the injured tissue [25].

Pulsed electromagnetic fields (PEMF) represent the modality with the longest clinical track record within this group of interventions and have been investigated in osteoarthritis, tendinopathies, bone healing, and low back pain [26,27]. Available experimental evidence indicates that PEMF can modulate the activity of adenosine A2A and A3 receptors, decrease the production of pro-inflammatory cytokines, attenuate the activation of NF-κB and MAPK pathways, promote angiogenesis, and stimulate chondrogenesis, osteogenesis, and extracellular matrix remodeling [28,29,30]. However, the clinical translation of these mechanisms continues to show heterogeneous results. While some studies have reported clinically relevant pain reductions and functional improvements, others have found modest benefits or a lack of differences compared to control interventions [25,31].

**Figure 1:**
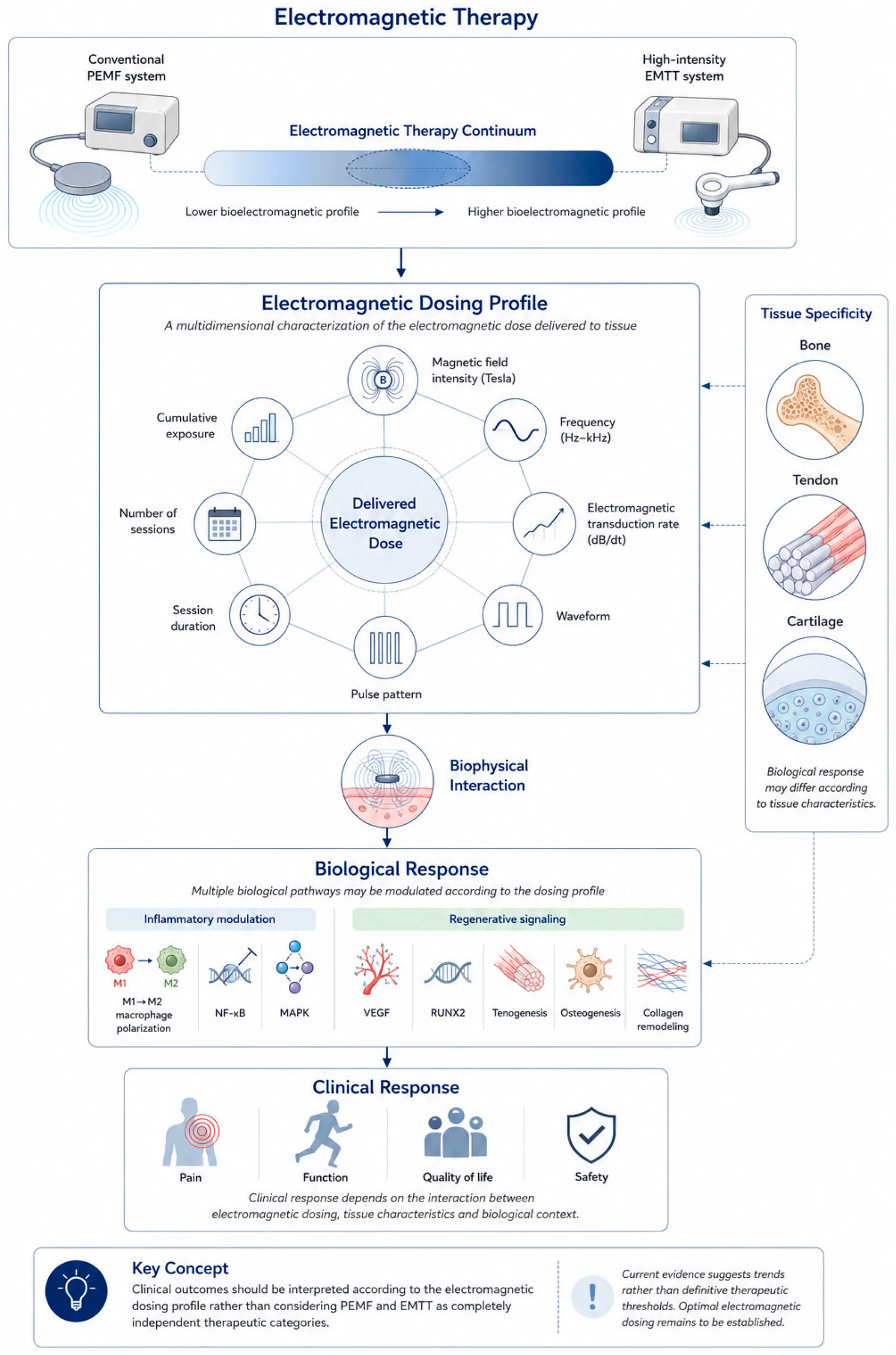
Conceptual framework of electromagnetic dosing determinants in chronic musculoskeletal disorders. This illustration was developed by the authors with assistance from ChatGPT (GPT-5.5, OpenAI) and underwent subsequent author review and refinement [46].

This variability likely does not reflect an inherent inconsistency in the technology, but rather the considerable diversity of physical parameters employed across studies [32]. Protocols that differ widely in magnetic field intensity, frequency, waveform, pulse duration, exposure time, and cumulative dose coexist under the umbrella term “PEMF.” From a biophysical perspective, these differences represent distinct biological stimuli; therefore, equivalent responses should not be expected [27,33,34]. Several experimental and translational reviews have highlighted the existence of “therapeutic windows” or dose-dependent effects, in which small modifications of electromagnetic parameters can significantly alter the magnitude and even the direction of the biological response [25,35,36]. Consequently, interpreting PEMF as a uniform intervention may lead to oversimplified conclusions and hinder the identification of protocols with the greatest therapeutic potential (Figure 1) [32].

Within the evolution of electromagnetic therapies, extracorporeal magnetotransduction therapy (EMTT) has emerged as a modality with distinct biophysical characteristics compared to conventional PEMF [37]. Unlike the latter, EMTT employs high-intensity pulsed electromagnetic fields, frequencies in the kilohertz range, and a significantly higher transduction rate, which allows for faster variations in the magnetic field [9,38] and, potentially, a different interaction with biological tissues [9]. Crucially, this physical distinction translates into a significant difference in biomechanical tissue penetration. While conventional PEMF systems frequently experience rapid spatial decay that limits their inductive capability in deeper structures, the high intensity and rapid transduction rate of EMTT generate a stronger, more sustained inductive coupling. This enables the effective delivery of therapeutic energy densities to high-resistance tissues—such as subchondral bone and deep intramuscular planes—that are typically beyond the optimal reach of low-intensity PEMF [37]. From this perspective, EMTT does not merely represent a quantitative intensification of the electromagnetic stimulus, but a qualitative modification of its physical characteristics, capable of activating distinct cellular responses [37,39].

Available preclinical evidence supports this hypothesis. Experimental studies have shown that EMTT stimulates the activity of human bone marrow-derived mesenchymal stem cells without evidence of cytotoxic effects, increasing the secretion of factors related to bone regeneration and angiogenesis [38]. Similarly, an increase in cell proliferation and migration, a decrease in markers associated with senescence, and the upregulation of tenogenic genes such as *Scleraxis* and Tenomodulin have been observed in human tenocytes, alongside increased expression of types I and III collagen—changes consistent with a more favorable phenotype for tendon repair [40]. In bone healing models, EMTT has also been associated with higher expression of osteogenic regulators, including *RUNX2*, and accelerated mineralization of the extracellular matrix, findings that support a potential pro-regenerative effect on bone tissue [37]. Nevertheless, available clinical evidence remains limited and presents variable results across different musculoskeletal entities. Randomized clinical trials have reported clinically relevant pain reductions and functional improvements in patients with non-specific low back pain and rotator cuff tendinopathy [41,42]. In contrast, a recent pilot trial on chronic myofascial pain syndrome confirmed the feasibility and safety of the intervention but found no significant differences compared to sham treatment regarding pain, functional interference, depressive symptoms, or global perception of change [43]. Viewed in isolation, these investigations do not allow for determining whether the observed variability stems from inherent differences between EMTT and PEMF or, conversely, from the extensive heterogeneity of the electromagnetic parameters employed [9], the populations studied, and the clinical contexts in which both technologies have been evaluated [26].

Consequently, the available literature often continues to interpret electromagnetic fields as a relatively homogeneous intervention [26], despite the fact that differences in intensity, frequency, and transduction rate represent potentially distinct biophysical stimuli [44]. The current lack of standardized dosing guidelines fosters an empirical approach to the administration of PEMF and EMTT; this not only hinders the reproducibility of clinical outcomes but also causes confusion among healthcare professionals when prescribing evidence-based treatments. This situation underscores the urgent need for unified international protocols to transform the use of these technologies from an empirical practice into a standardized intervention. To the best of our knowledge, no systematic review has specifically integrated these parameters as a primary axis of analysis to explore their influence on clinical response [45].

Therefore, the objective of this systematic review was to analyze contemporary advances (2015–2026) regarding the efficacy and safety of PEMF and EMTT in adults with chronic musculoskeletal injuries by performing an integrated analysis of how dosage parameters—including intensity, frequency, waveform, pulse duration, and cumulative dose—influence clinical outcomes, with the ultimate goal of identifying patterns that support a more rational and evidence-based electromagnetic prescription.

## 2. Methods

### 2.1. Study Design and Registration

This systematic review was conducted in accordance with the Preferred Reporting Items for Systematic Reviews and Meta-Analyses (PRISMA 2020) [47] statement and its extension for protocols (PRISMA-P) [48]. Due to the clinical, methodological, and electromagnetic dosing heterogeneity identified among the studies, results were synthesized using the Synthesis Without Meta-analysis (SWiM) framework in cases where quantitative data pooling was inappropriate or methodologically unjustifiable [49].

### 2.2. Eligibility Criteria

Randomized controlled trials (RCTs), quasi-experimental studies (with or without a control group), as well as case reports and case series providing relevant information on clinical application, safety, or hypothesis generation regarding Extracorporeal Electromagnetic Transduction Therapy (EMTT) and Pulsed Electromagnetic Fields (PEMF) were included. Conversely, preclinical studies (in vitro and animal models), systematic reviews, narrative reviews, meta-analyses, editorials, letters to the editor, gray literature, and conference abstracts were excluded.

The population of interest consisted of individuals aged ≥18 years with a clinical or imaging diagnosis of musculoskeletal disorders. This definition was broadened to encompass both acute conditions (such as fractures) and chronic pathologies, as the analyzed evidence demonstrated that PEMF and EMTT are applied across both bone healing and pain management contexts. Specifically, patients with knee osteoarthritis, chronic non-specific low back pain, myofascial pain syndrome, lateral or medial epicondylopathy, tendinopathies, confirmed rotator cuff injuries, delayed bone healing (delayed union), non-union (pseudoarthrosis), and other conditions amenable to electromagnetic treatment were included. Research conducted exclusively on pediatric populations or patients with systemic diseases that could significantly alter biological response—such as active rheumatoid arthritis, severe neurological disorders, or uncontrolled diabetes mellitus—was excluded.

The interventions of interest were EMTT and PEMF modalities. To analyze the influence of dosage on clinical outcomes, the following physical parameters were systematically documented: device used; maximum magnetic field intensity; frequency; field variation or transduction rate (dB/dt); pulse shape and pattern; session duration; total number of sessions; interval between applications; and cumulative exposure. Comparators included sham procedures, placebo, conventional physical therapy, therapeutic exercise, pharmacological treatment, or standard care. Finally, only studies conducted in clinical settings and published in English or Spanish were considered.

### 2.3. Outcome Measures

Primary outcome measures were pain intensity and functional recovery. Pain intensity was recorded using validated scales, primarily the Visual Analogue Scale (VAS) and the Numeric Rating Scale (NRS). Functional recovery was assessed using an organized categorical framework to facilitate cross-study comparison, encompassing three domains: (1) Patient-Reported Outcome Measures (PROMs) reflecting disability and activities of daily living (e.g., WOMAC, DASH, ODI); (2) performance-based tests for objective capacity (e.g., Timed Up and Go, gait speed); and (3) biomechanical/impairment variables (e.g., Range of Motion and muscle strength dynamometry).

Secondary outcomes included health-related quality of life (SF-36, SF-12, EQ-5D) and treatment safety (adverse events). To facilitate comparison, results were grouped by follow-up period into: short-term (≤12 weeks), medium-term (>12 weeks to 6 months), and long-term (>6 months). The narrative analysis was organized by intervention type, clinical condition, and dosing parameters.

### 2.4. Information Sources and Search Strategy

A systematic search was performed in PubMed/MEDLINE [50], the Cochrane Central Register of Controlled Trials (CENTRAL) [51], and ScienceDirect [52], covering the period from 1 January 2015 to 31 December 2025. This interval allowed for the capture of contemporary evidence on EMTT and PEMF using devices representative of current clinical practice.

**Figure 2:**
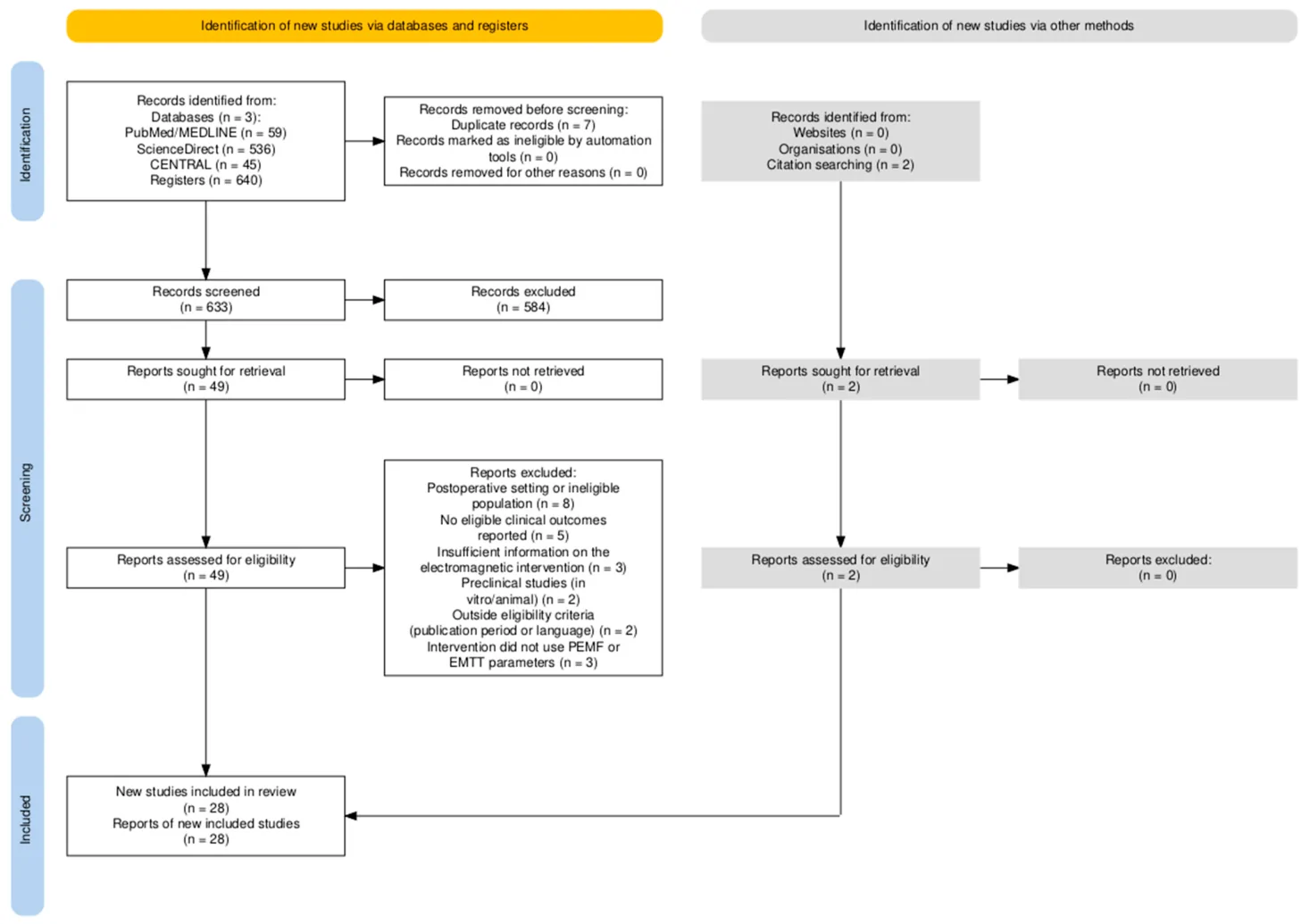
PRISMA flow diagram of the study selection process [53].

Search strategies combined controlled vocabulary (MeSH) and free-text terms. The 2015–2025 timeframe was purposively selected to ensure technological parity. Modern PEMF and particularly EMTT devices utilize physical parameters (e.g., higher intensities and fast magnetic field variation rates [dB/dt]) that differ substantially from technologies used in previous decades. Excluding older literature prevented a critical bias of technological heterogeneity, ensuring that the dose–response synthesis is applicable to contemporary clinical practice. In addition to electronic database searches, we prospectively planned to supplement our systematic review with citation searching (screening the reference lists of included studies) to identify potentially relevant publications that might have been omitted by the primary database search strategy. This snowballing approach was employed to ensure comprehensive coverage and was integrated into our screening process as detailed in Figure 2. Full database-specific search strings are provided in Supplementary File S1.

### 2.5. Study Selection

All retrieved records were imported into Mendeley Reference Manager (version 2.114.0; Elsevier, Amsterdam, Netherlands) for duplicate identification and removal. Subsequently, the selection process was conducted in two consecutive stages: (1) screening of titles and abstracts and (2) full-text review of potentially eligible studies. Both phases were performed independently by two reviewers (ARA and JSAA). Discrepancies were resolved through discussion and, when necessary, with the involvement of a third reviewer (VARH). During the full-text evaluation, reasons for the exclusion of studies that did not meet the pre-established eligibility criteria were documented.

### 2.6. Data Extraction

Data extraction was performed using a structured, pre-piloted form developed in Microsoft Excel (Microsoft 365). Two reviewers (ARA and ILM) independently extracted information regarding the general characteristics of each study: author, year of publication, country, methodological design, recruitment period, sample size, population characteristics, diagnostic criteria, intervention, comparator, assessed outcomes, follow-up periods, quantitative results, adverse events, funding sources, and conflicts of interest.

Since the primary objective of this review was to analyze the influence of electromagnetic dosing, the physical characteristics of each protocol were also systematically recorded: device used, peak magnetic field intensity, frequency, waveform, pulse pattern, field variation rate (dB/dt) when reported, session duration, number of applications, inter-session interval, and cumulative exposure. When critical information for the analysis was unavailable in the original manuscript, attempts were made to contact the corresponding authors to request additional data.

### 2.7. Risk of Bias Assessment

Risk of bias was independently assessed by two reviewers (ARA and ILM). Randomized clinical trials were evaluated using the Cochrane Risk of Bias 2 (RoB 2) tool, considering domains related to the randomization process, deviations from intended interventions, missing outcome data, measurement of the outcome, and selection of the reported result.

Non-randomized studies were evaluated using the Risk of Bias in Non-Randomized Studies of Interventions (ROBINS-I) tool, which examines seven domains related to confounding, participant selection, classification of interventions, deviations from intended interventions, missing data, measurement of outcomes, and selection of reported results.

Case reports and case series were evaluated using the Joanna Briggs Institute (JBI) Critical Appraisal Checklists for Case Reports and Case Series, adapted to assess clarity in clinical description, diagnostic process, intervention characteristics, reported outcomes, limitations, and the potential for generalizability of the findings. Each study was evaluated independently, with identified methodological strengths and limitations documented narratively.

### 2.8. Data Synthesis

Due to clinical, methodological, and electromagnetic dosing heterogeneity, a meta-analysis was not feasible for most outcomes. Consequently, results were synthesized using a narrative approach in accordance with the SWiM framework recommendations [49].

The evidence was organized hierarchically according to intervention type (EMTT, PEMF, and combined therapies), clinical condition, outcome domain (pain, function, quality of life, and safety), follow-up time, risk of bias, and reported dosing parameters. When possible, continuous results were described using absolute values and the changes observed between baseline and final assessments. In the absence of conditions for a valid quantitative comparison, the evidence was integrated through narrative synthesis, emphasizing the direction, magnitude, and consistency of the effects observed across the studies.

Furthermore, to evaluate the long-term sustainability of the clinical benefits, a specific quantitative analysis of the retention effect was conducted for studies that reported both immediate post-intervention data (end of therapy) and medium-to-long-term follow-up scores. A retention delta (Δ) was calculated as the numerical difference between the final follow-up score and the immediate post-treatment score, allowing for the objective assessment of whether initial clinical gains were maintained, amplified, or lost after the cessation of the electromagnetic stimulus.

### 2.9. Data Visualization and Software

To ensure transparency and reproducibility, we specified the tools utilized for data synthesis and graphic generation. The PRISMA 2020 flow diagram was generated following the official PRISMA 2020 guidelines using the prisma2020 R package and Shiny app [53]. Quantitative data synthesis and statistical visualization were performed using GraphPad Prism (version 9.5.1; GraphPad Software, San Diego, CA, USA). Conceptual illustrations and schematic figures were developed with the assistance of the generative AI model ChatGPT (GPT-5.5; OpenAI, San Francisco, CA, USA) [46], and subsequently reviewed and finalized by the authors to ensure scientific accuracy, clinical coherence, and strict alignment with the analyzed evidence base.

## 3. Results

### 3.1. Literature Search and Selection Results

The literature search identified 635 unique records from electronic databases. To ensure comprehensive coverage, a manual citation search was performed concurrently, identifying two additional records. Of the 637 total records identified, 7 duplicates were removed, leaving 630 unique records to be screened by title and abstract. As detailed in the PRISMA flow diagram (Figure 2), the two records identified via manual searching were integrated into the eligibility workflow alongside the database results. Both underwent full-text review, met eligibility criteria, and were included in the final qualitative synthesis, contributing to the 28 studies analyzed.

The included evidence comprised 18 clinical trials [41,42,43,54,55,56,57,58,59,60,61,62,63,64,65,66,67,68], 2 quasi-experimental studies [69,70], and 8 case reports (Table 1) [71,72,73,74,75,76,77,78]. Although the case reports represent a lower level of evidence, they were retained to provide complementary data on feasibility, safety, and functional recovery in specific clinical scenarios—particularly regarding return to work and sports, which are infrequently documented in comparative studies.

Altogether, the included studies analyzed 1049 participants, of whom 376 were male and 549 were female (sex was not reported for 124 participants). The mean age was 43.64 ± 13.58 years. The investigations covered 27 distinct musculoskeletal conditions and evaluated interventions based on PEMF and EMTT, employing a wide variety of devices, electromagnetic parameters, comparators, and associated rehabilitation programs (Table 1). This clinical, methodological, and technological diversity justified conducting a structured narrative synthesis following the SWiM recommendations.

The most frequently investigated clinical conditions were knee osteoarthritis [61,63,64,65,68], acute or delayed union fractures [60,76,77,78], soft tissue injuries [55,58,66,69,74], and non-specific low back pain [41,56,57,59,67]. Studies were also identified regarding non-unions [71,72,73,75], myofascial pain syndrome [43,62], Achilles tendinopathy [70], rotator cuff tendinopathy [42], and lateral epicondylopathy [54], collectively forming a broad spectrum of conditions characterized by impaired tissue repair, persistent inflammation, or chronic musculoskeletal pain.

To conduct a systematic analysis of the evidence, a database was constructed that consolidated 361 individual outcome records distributed across 10 main clinical domains (Table 2). The domains of function (41.8%) and pain (26.6%) accounted for more than two-thirds of the extracted records, followed by quality of life (9.1%) and safety and feasibility (6.4%). The remaining domains comprised outcomes related to imaging and tissue healing, mental health, electrophysiology, patient global perception, inflammation and biomarkers, and clinical symptoms. This structure allowed for the synthesis of evidence not only by clinical condition and intervention type but also by the physical parameters employed in the various EMTT and PEMF protocols. This approach facilitated the identification of clinical response patterns across different technologies and dosing regimens.

### 3.2. Risk of Bias and Methodological Quality

**Figure 3:**
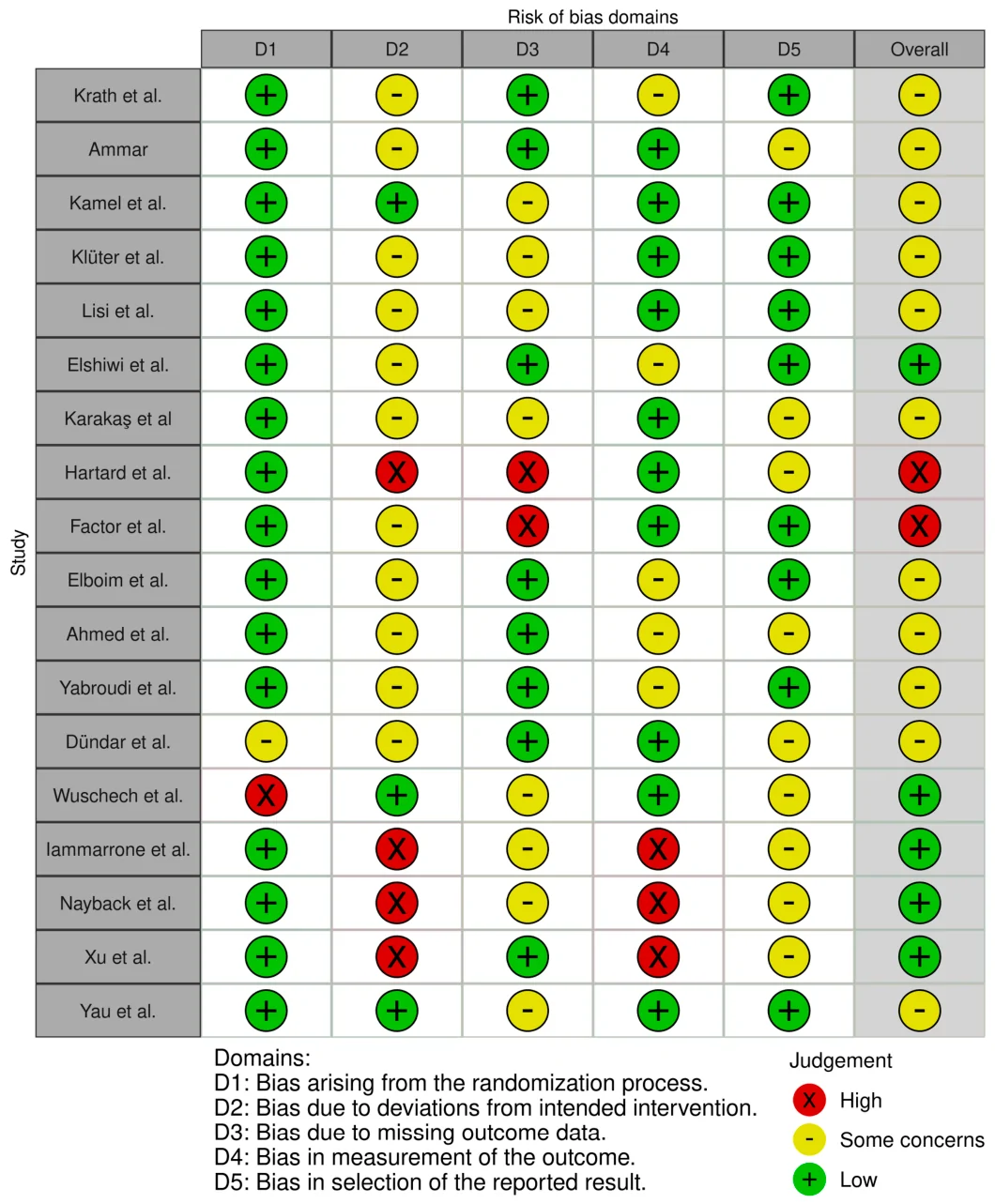
Risk of bias summary (RoB 2) for the included randomized controlled trials [79].

The methodological quality of the included studies varied and was closely linked to the research design. The 18 randomized controlled trials were assessed using the RoB 2 tool; most were judged to have “some concerns,” while a smaller number were classified as “high risk of bias” (Figure 3). The domains that most frequently contributed to these judgments were the randomization process and deviations from intended interventions. These ratings were primarily due to insufficient information regarding allocation concealment procedures, blinding of participants and personnel, or adherence to intervention protocols. Conversely, the domains related to missing outcome data and measurement of the outcome predominantly showed a low risk of bias.

**Figure 4:**
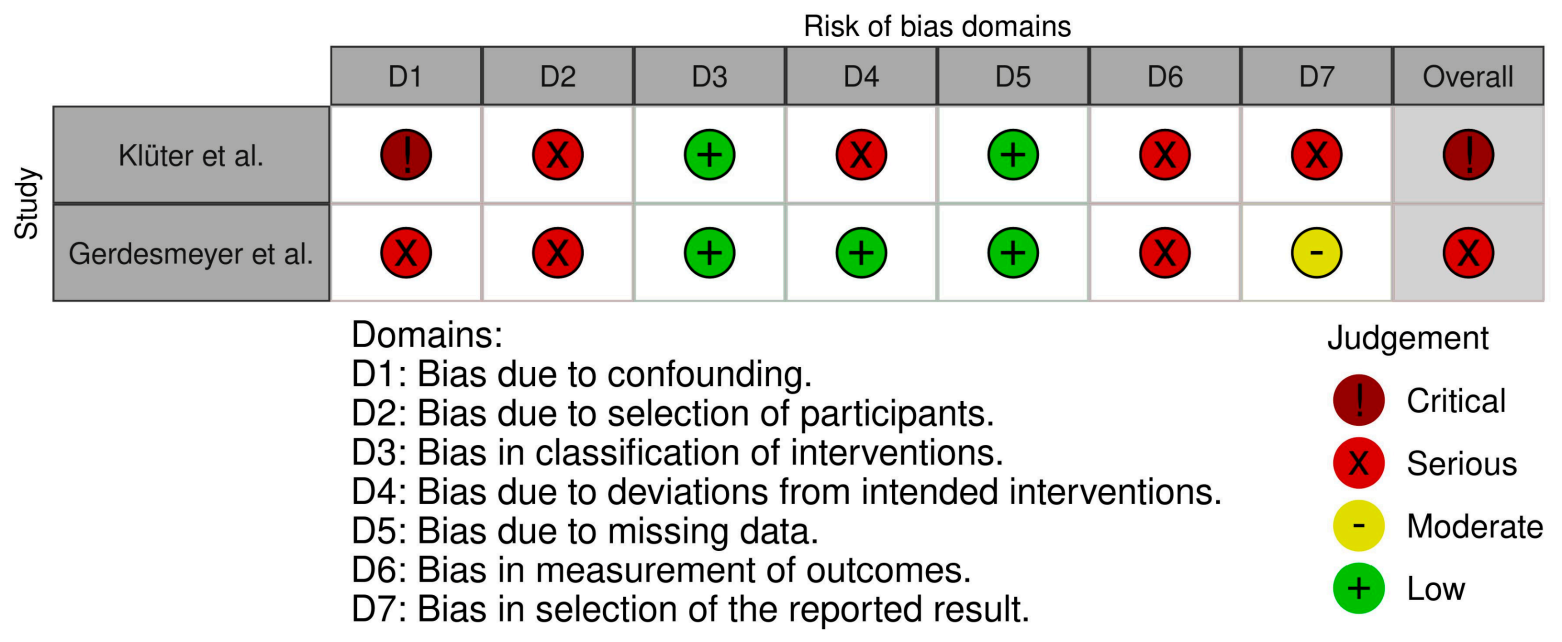
Risk of bias assessment in quasi-experimental studies using the ROBINS-I tool [79]. Produced with the robvis visualization tool.

The two quasi-experimental studies, assessed using the ROBINS-I tool, presented an overall risk of bias classified as “serious” and “critical,” respectively (Figure 4). The primary sources of bias were associated with confounding, participant selection, and deviations from intended interventions—methodological limitations inherent to these types of designs.

The eight case reports, assessed using the Joanna Briggs Institute (JBI) critical appraisal tool, demonstrated adequate descriptive methodological quality. All reports clearly described patient clinical characteristics, the diagnostic process, the interventions applied, and the observed outcomes, while also addressing the inherent limitations of the study design. Overall, these studies met approximately 80% of the critical appraisal criteria. This supports their utility as descriptive evidence for documenting the clinical feasibility and safety of these technologies, although they do not allow for causal inferences.

### 3.3. Pain

Pain was the second most frequently assessed clinical domain in this review, representing 96 individual records (26.6% of total outcomes) (Table 2). Pain intensity was primarily quantified using the Visual Analogue Scale (VAS) and Numeric Rating Scale (NRS). As detailed qualitatively in Table 3, both extracorporeal magnetotransduction therapy (EMTT) and pulsed electromagnetic fields (PEMF) were generally associated with pain reduction. However, quantitative analysis of the extracted data revealed that the magnitude of the clinical effect varied considerably according to the research design and the specific electromagnetic dosing profile (intensity, cumulative dose, and transduction rate).

In randomized controlled trials (RCTs) evaluating high-intensity EMTT (80 mT, 3 Hz, 20 min/session, 8 sessions; 12,800 mT·min cumulative dose), substantial pain reductions were consistently reported. In patients with non-specific low back pain, EMTT combined with conventional care progressively decreased pain intensity from 5.8 ± 0.82 to 2.05 ± 1.01 at 18 weeks (a 3.75-point reduction), surpassing the 2.66-point reduction observed in the control group [41]. When combined with extracorporeal shock wave therapy (ESWT) for rotator cuff tendinopathy, EMTT yielded an absolute VAS reduction of 5.43 points (from 6.16 ± 0.90 to 0.73 ± 0.25), compared with a 4.12-point reduction in the ESWT-plus-sham group [42]. However, response magnitudes varied by pathology; in chronic myofascial pain syndrome, EMTT achieved a more modest mean NRS reduction (from approximately 6.5 to 4.3 points) that did not demonstrate clinically meaningful superiority over sham at the six-month follow-up [43].

Clinical trials evaluating PEMF demonstrated a broader spectrum of quantitative responses, highly dependent on the physical parameters employed. High-intensity and high-cumulative-dose protocols reported the largest absolute pain reductions. For example, high-energy PEMF (HE-PEMF, 50 mT) reduced back pain intensity from 6.50 ± 1.41 to 1.36 ± 0.90 (−5.14 points) after only three sessions, compared with a −2.59-point change in the sham group [59]. In post-natal carpal tunnel syndrome, a 12-session PEMF protocol (8 mT, 50 Hz; 2880 mT·min) achieved a striking 4.93-point VAS reduction, which was significantly superior to the 1.30-point reduction produced by therapeutic ultrasound [55]. Furthermore, in lateral epicondylopathy, PEMF combined with exercise decreased VAS scores from 6.23 to 2.88 (a 3.35-point reduction), outperforming both microcurrents and exercise alone [54]. In knee osteoarthritis, high-intensity protocols (105 mT) produced significant pain reductions versus placebo [65], and the combination of PEMF with platelet-rich plasma (PRP) reduced VAS scores from 8.44 ± 0.81 to 2.75 ± 0.58 at 12 weeks, quantitatively surpassing isolated treatments [68]. In acute distal radius fractures, a portable PEMF device (0.05–0.5 mT) applied continuously for up to six weeks significantly improved the PRWE pain subscale compared to sham [60].

Conversely, RCTs utilizing lower magnetic field intensities or generating low cumulative doses frequently reported marginal inter-group differences. For instance, in bilateral knee osteoarthritis, a 20-session protocol utilizing 0.1 mT (40 mT·min cumulative dose) yielded a 40 mm reduction on a 100 mm VAS, which was clinically comparable to the 30 mm reduction observed in the sham group [64]. Similarly, in chronic low back pain, a 12-session protocol using 2 mT (480 mT·min) reduced VAS scores from 7.66 to 5.24 (−2.42 points), providing no additional quantitative benefit over the −3.94-point change achieved by the control group [57]. Trials employing 2 mT for chronic neck pain [58] and 5 mT for mild-to-moderate knee osteoarthritis [63] also demonstrated within-group pain reductions that were statistically indistinguishable from their respective sham or active exercise comparators. Other low-intensity portable devices for home application in non-specific low back pain showed clinically relevant improvements but no significant absolute differences between PEMF and sham arms [56,67].

Quasi-experimental designs evaluating EMTT (80 mT) documented robust quantitative pain reductions, supporting the trends observed in the RCTs. In patients with chronic aseptic osteitis pubis, just four EMTT sessions rapidly reduced VAS scores from 7.5 ± 1.0 to 0.5 ± 0.72 within two weeks, achieving a mean score of 0.0 at the three-month follow-up [69]. In midportion Achilles tendinopathy, eight sessions of EMTT decreased pain intensity from 6.9 ± 1.3 to 3.6 ± 2.0 (−3.3 points), showing a greater absolute magnitude of improvement than the −1.7-point reduction observed in patients treated solely with heel cushions [70].

Finally, descriptive data from case reports consistently documented complete or near-complete pain resolution across various complex clinical scenarios. In non-unions of the scaphoid, fifth metacarpal, humeral shaft, and hamulus ossis hamati, EMTT (frequently combined with ESWT) was associated with progressive pain relief that paralleled radiographic bone consolidation [71,72,73,75]. Similar clinical trajectories, with complete resolution of localized pain and return to high-impact sports, were reported in patients treated for medial meniscal tears [74], clavicle fractures [76], and femoral neck stress fractures [77].

### 3.4. Function

Functionality was the most frequently assessed clinical outcome, comprising 151 individual records (41.8% of the total outcomes) (Table 2). To address the methodological diversity of these assessments, functional recovery outcomes were categorized by specific functional domains. Patient-Reported Outcome Measures (PROMs) were widely utilized to capture subjective functional gains (e.g., ODI, WOMAC, and DASH). These were frequently complemented by performance-based tests (e.g., TUG, functional walking assessments) and biomechanical variables (e.g., grip strength and goniometry for joint ROM), allowing for a multidimensional evaluation of recovery. As summarized qualitatively in Table 4, the narrative synthesis revealed a consistent trend toward functional improvement across all domains; however, the quantitative data demonstrated that the magnitude of recovery was highly dependent on the electromagnetic dosing profile (intensity and cumulative exposure).

In randomized controlled trials (RCTs), high-intensity EMTT protocols (80 mT, 3 Hz, 20 min/session) consistently elicited robust functional gains. In non-specific low back pain, eight sessions of EMTT (12,800 mT·min) decreased the Oswestry Disability Index (ODI) from 53.48 ± 9.85 to 20.82 ± 8.59 at 18 weeks—a substantial 32.66-point improvement—which was clinically superior to the 22.57-point reduction achieved by conventional physiotherapy [41]. Similarly, when combined with extracorporeal shock wave therapy (ESWT) for rotator cuff tendinopathy, this EMTT protocol increased the Constant-Murley Score from 59.44 ± 12.50 to 93.10 ± 0.69 (a 33.66-point gain), compared to a 22.08-point improvement in the group treated with ESWT alone [42]. In contrast, EMTT applied to chronic myofascial pain syndrome yielded functional improvements that failed to establish clinically meaningful superiority over sham treatment [43].

PEMF interventions exhibited a wide variance in functional recovery that closely aligned with the prescribed dosage. High-cumulative-dose protocols reported the most prominent differences versus control groups. In acute distal radius fractures, continuous application of a portable PEMF device (0.05–0.5 mT, 24 h/day) significantly accelerated recovery; at 12 weeks, grip strength reached 14.22 ± 2.67 kg compared to 8.25 ± 2.19 kg in the sham group, while wrist flexion measured 65° versus 33° [60]. In lateral epicondylopathy, a 12-session PEMF protocol (30 min/session) combined with exercise reduced the Disabilities of the Arm, Shoulder and Hand (DASH) score by 8.80 points and increased grip strength by 5.83 kg, quantitatively outperforming the 4.94-point DASH reduction and 2.51 kg strength increase achieved by exercise alone [54]. In knee osteoarthritis, a 10 mT PEMF protocol reduced the total Western Ontario and McMaster Universities Osteoarthritis Index (WOMAC) score by 18.9 points, compared to a 10.1-point reduction in the low-level laser therapy group [61]. High-intensity PEMF (105 mT) also produced significant functional improvements over placebo [65], and extended home-based applications (240 min/day) yielded greater long-term functional gains in patellofemoral pain syndrome than exercise alone [66].

Conversely, RCTs utilizing lower magnetic field intensities or delivering a low cumulative dose often reported functional gains that were statistically indistinguishable from sham or conventional treatments. In knee osteoarthritis, protocols employing 0.1 mT [64] and 5 mT [63] documented functional improvements that provided no clinically relevant advantage over sham procedures or progressive resistance exercise, respectively. Similarly, in chronic neck pain, a 15-session protocol using 2 mT improved the Neck Pain Disability Scale from 57.5 ± 22.1 to 33.7 ± 22.9, a progression comparable to that observed in the sham group [58]. In chronic low back pain, adjunctive PEMF at 2 mT did not provide additional functional benefit beyond conventional rehabilitation [57].

To account for the profound heterogeneity among active co-interventions and control groups, Table 5 systematically categorizes the specific exercise modalities employed across the included trials. These control interventions varied significantly in their primary therapeutic objectives, ranging from immediate analgesia to structural tissue remodeling.

Quasi-experimental designs evaluating EMTT corroborated the functional trends observed in the RCTs. In chronic Achilles tendinopathy, eight sessions of EMTT (80 mT) improved the Roles-Maudsley Score from 3.61 ± 0.50 to 2.57 ± 0.92; this reflected a greater absolute magnitude of recovery than the control group treated with heel cushions, which shifted from 3.68 ± 0.48 to 2.92 ± 0.78 [70]. Furthermore, a rapid, progressive return to unrestricted sports participation and full function was documented in patients with chronic osteitis pubis following EMTT [69].

Case reports and series provided supplementary descriptive evidence of functional progression in highly complex clinical scenarios. Across various non-unions and stress fractures, combined interventions (frequently EMTT + ESWT) were associated with the full restoration of joint range of motion, recovery of grip strength, and clinically relevant improvements in functional scales [71,72,73,74,75,76,77,78]. Although these uncontrolled observations preclude causal inferences, they consistently documented the clinical feasibility of achieving full functional recovery and facilitating a return to physically demanding work and competitive sports following high-energy electromagnetic interventions.

### 3.5. Quality of Life

Health-related quality of life (HRQoL) was the third most frequently assessed outcome, comprising 33 individual records (9.1% of total outcomes) (Table 2). Evaluations relied predominantly on the SF-36, SF-12, and KOOS-QoL instruments. As summarized qualitatively in Table 6, the effects of electromagnetic interventions on quality of life were heterogeneous, reflecting the diversity of instruments utilized and the disparities in physical parameters across specific dosing protocols.

Among the randomized controlled trials evaluating EMTT, evidence regarding broad quality-of-life domains remains limited. The pilot trial by Yau et al. [43] applied a high-intensity protocol (80 mT, 3 Hz, 20 min sessions; approximate cumulative dose of 12,800 mT·min) in patients with chronic myofascial pain syndrome. Although several SF-36 domains showed within-group numerical improvements relative to baseline, the absolute differences compared to the sham group were modest and failed to demonstrate clinically meaningful superiority during the follow-up period.

The majority of the HRQoL evidence focused on PEMF interventions. The most prominent quantitative benefit was observed in a high-exposure protocol for acute distal radius fractures, where Factor et al. [60] evaluated a portable PEMF device applied continuously (0.05–0.5 mT, 10 Hz) 24 h daily. At 12 weeks, the physical component summary (PCS) of the SF-12 reached 47 points in the treated group versus 36 points in the sham group (*p* = 0.005). However, the mental component summary (MCS) showed no significant differences between the study arms.

Conversely, protocols utilizing lower magnetic field intensities or shorter cumulative exposures reported HRQoL improvements that were statistically indistinguishable from control groups. In patients with chronic neck pain, Karakaş and Gök [58] employed a protocol of 2 mT for 15 sessions (approximate cumulative dose of 600 mT·min). Both groups experienced numerical improvements across several SF-36 domains—including physical functioning, physical role, energy/vitality, and bodily pain—but the differences between the PEMF and sham groups were not statistically significant at the conclusion of the intervention. Similarly, Nayback-Beebe et al. [67] noted improvements in the physical health domains of the SF-36 following PEMF treatment for chronic low back pain, yet observed no clinically meaningful differences over standard care, while reporting an increase in anxiety scores during follow-up. In mild-to-moderate knee osteoarthritis, Yabroudi et al. [63] reported that while the KOOS-QoL subscale improved following 24 sessions of a 5 mT PEMF protocol, the addition of PEMF to a progressive resistance exercise program provided no additional quantitative benefit over exercise alone.

### 3.6. Quantitative Retention Effect (Post-Intervention to Final Follow-Up)

An analysis of post-intervention follow-up periods identified 12 studies that evaluated the retention effect of electromagnetic therapies. These were categorized into two distinct groups: quantitative studies (n = 7) providing measurable changes (deltas) and descriptive case reports/series (n = 5) documenting clinical milestones. To quantify the durability of treatment effects, a retention delta (Δ) was calculated for the seven studies reporting complete numerical data at both the immediate post-intervention timepoint and the final follow-up (Table 7). The remaining five studies provided descriptive follow-up data focused on clinical and radiographic consolidation. The quantitative data characterize the trajectory of clinical scores following the cessation of electromagnetic therapy.

### 3.7. Adverse Events Profile

As prespecified in the secondary outcome measures, treatment safety and tolerability were systematically evaluated across all included trials. Overall, both EMTT and PEMF interventions demonstrated an excellent safety profile. Table 8 summarizes the specific adverse events (AEs) documented in the six trials that explicitly reported safety data [41,42,43,56,68,70]. Notably, no serious, permanent, or life-threatening adverse events were reported among the 1049 participants evaluated in this systematic review. All documented treatment-related AEs were restricted to local, superficial tissue reactions (such as transient erythema or mild petechiae) or transient post-treatment exacerbations of pain; these were classified as mild, strictly self-limiting, and resolved spontaneously without requiring therapy discontinuation.

## 4. Discussion

The present systematic review synthesized available clinical evidence regarding the effects of extracorporeal magnetotransduction therapy (EMTT) and pulsed electromagnetic fields (PEMF) in adults with chronic musculoskeletal injuries. Collectively, the results demonstrate that both modalities are associated with improvements in pain and functional recovery, though effects on quality of life remain less definitive [26,45]. However, the magnitude and consistency of these clinical effects varied considerably across studies. While our initial hypothesis posited that the electromagnetic dosing profile (intensity, frequency, and cumulative dose) plays a pivotal role in this heterogeneity, a rigorous interpretation requires a multidimensional analysis [32,80]. The observed clinical responses are not solely the product of electromagnetic physics; they are equally modulated by the underlying pathology, the nature of active co-interventions, the choice of active comparators, and the methodological quality of the available evidence.

This physical reality has profound implications for treatment efficiency and posology. Because higher transduction rates achieve cellular activation thresholds through a larger biological stimulation window per pulse, significantly fewer therapeutic sessions are required. This aligns with our findings, where high-transduction EMTT and high-energy PEMF protocols frequently elicited significant clinical changes within short protocols of just 6 to 8 sessions [41,59,69]. In contrast, low-intensity PEMF devices often required extended protocols of 15 to 20 sessions, or continuous daily applications, to achieve comparable functional outcomes [56,60,64].

Furthermore, understanding the spatial propagation of the magnetic field is essential to elucidating clinical efficacy, highlighting a critical biomechanical difference in tissue penetration. According to the inverse-square law, magnetic field intensity decays exponentially as the distance from the applicator to the target tissue increases [33]. From a clinical dosimetry perspective, a gap of just 1 to 2 cm between the applicator and the skin can drastically reduce the effective transfer rate (dB/dt) reaching the target tissue. Therefore, optimal clinical positioning—ensuring direct contact and a perpendicular (90°) angle of incidence—is not merely a matter of patient comfort, but a critical determinant of the effective ‘absorbed dose’ [81]. While low-intensity PEMF devices may be highly effective for superficial structures—such as acute distal radius fractures [60]—where the target tissue is only millimeters away, deeper anatomical structures, such as the lumbar spine or deep intramuscular trigger points, require a substantially higher initial electromagnetic output. High-intensity systems (EMTT) compensate for this spatial decay through an elevated rate of field variation, generating a deeper inductive coupling that allows effective, non-invasive mechanotransduction in high-resistance, deep musculoskeletal tissues [9,37].

While the electromagnetic dosing hypothesis provides a strong biophysical foundation, relying on it exclusively overlooks other critical variables that explain the heterogeneity of the present results. The efficacy of electromagnetic therapies appears to be heavily dependent on the pathophysiological mechanisms of the treated condition. EMTT and PEMF demonstrated their greatest efficacy in structural and mechanically driven pathologies, such as non-unions, osteoarthritis, and degenerative tendinopathies. In these conditions, the induced fields modulate peripheral local processes, including macrophage polarization (M1 to M2), angiogenesis, and osteogenic/tenogenic differentiation [13,25,82,83]. Conversely, in conditions where central sensitization or nociplastic mechanisms predominate (such as chronic myofascial pain syndrome and chronic non-specific neck pain), the clinical benefits of electromagnetic interventions frequently paralleled those of sham treatments [43,58]. Central sensitization involves the amplification of nociceptive processing within the central nervous system [84]. Because PEMF and EMTT primarily act on peripheral tissue repair, it is plausible that they lack the capacity to reverse centrally mediated chronic pain without concurrent multimodal psychological and pharmacological support, explaining the modest analgesic responses in these populations [85,86].

Regarding the consistency of these findings with the broader literature, the dose–response heterogeneity identified in this review aligns closely with previous systematic reviews evaluating PEMF. However, to move beyond a mere sequential enumeration of study outcomes, our narrative synthesis explicitly contrasted the clinical efficacy across different dosimetric profiles to identify potential ‘therapeutic windows’ [87]. Within the included randomized controlled trials (RCTs), a clear dosimetric dichotomy emerged: interventions employing higher magnetic field intensities (e.g., 50–105 mT) or generating high cumulative exposures consistently surpassed placebo and active controls in structural pathologies. In sharp contrast, protocols utilizing very low magnetic field intensities (e.g., 0.1 to 5 mT) or short cumulative exposure times frequently resulted in therapeutic failures, yielding clinical improvements that were statistically indistinguishable from sham devices or baseline exercise alone [57,63,64]. This dose–response pattern strongly corroborates the hypothesis that a critical minimum therapeutic threshold of tissue mechanotransduction must be met to trigger a significant biological repair response; falling below this amplitude window renders the electromagnetic intervention clinically ineffective.

Nevertheless, attributing the success of these RCTs solely to electromagnetic parameters (e.g., frequency or intensity) would be an oversimplification. The magnitude of the observed effects was equally modulated by the natural history of the lesions, symptom chronicity, and methodological quality. Pain and functional scales are highly susceptible to placebo effects, particularly in trials utilizing sophisticated medical devices—such as high-intensity EMTT systems—that engender significant patient expectations. This underscores why trials employing rigorous sham devices and strict allocation concealment frequently reported smaller between-group differences than unblinded or poorly controlled studies, emphasizing the necessity of high-quality trial designs to prevent the overestimation of treatment effects [56,64].

Crucially, the magnitude of clinical change was profoundly influenced by the specific exercise modality utilized in the control groups (Table 5). Grouping all active interventions under the umbrella of ‘conventional physical therapy’ obscures the fact that these protocols operate through fundamentally distinct biological pathways. For instance, in tendinopathies, isometric exercises primarily induce descending cortical inhibition, providing immediate analgesia without driving significant structural tissue remodeling [88]. In contrast, progressive resistance exercise (PRE) provides a profound mechanical load that directly stimulates tenocyte proliferation and extracellular matrix synthesis—the primary biological drivers of tissue remodeling [89]. Consequently, the perceived efficacy of EMTT or PEMF depends heavily on the mechanical baseline provided by the control intervention. When evaluated alongside robust PRE regimens, the magnetic field appears to act primarily as a biological adjuvant; in these cases, it may struggle to demonstrate statistical superiority because the robust physiological adaptations generated by the exercise itself leave a narrow margin for additional benefit [57,63]. Conversely, when the control group relies solely on isometrics or passive modalities, the active mechanical stimulus for remodeling is absent; in these scenarios, the regenerative capacity of EMTT or PEMF frequently manifests as a primary driver of structural and functional recovery.

A notable finding of this review was that functional recovery exhibited a more homogeneous and consistent trend than pain reduction. Function depends primarily on the mechanical capacity of the tissue, joint mobility, and progressive load tolerance, whereas the pain experience is a complex phenomenon influenced by neurophysiological, cognitive, and emotional factors, such as hypervigilance and kinesiophobia [4]. This observation is congruent with musculoskeletal rehabilitation principles, where the restoration of mobility and mechanical function frequently precedes the resolution of pain [90]. Therefore, functional recovery may represent a more sensitive indicator of the peripheral biological effects induced by electromagnetic fields during the early phases of rehabilitation.

Our quantitative evaluation of post-intervention follow-up data suggests a pathology-dependent retention pattern. In structural and mechanically driven conditions (e.g., fractures, non-unions, and early-stage osteoarthritis), the biological response triggered by the electromagnetic field appears to persist and potentially amplify over time. This is evidenced by the continuous improvements in calculated deltas for grip strength, Feller scores, and WOMAC values observed weeks or months after therapy cessation. This delayed, progressive improvement may be grounded in the modulation of the macrophage phenotype (M1-to-M2 transition), which promotes a prolonged regenerative environment characterized by anti-inflammatory cytokine release (e.g., IL-10) and enhanced collagen remodeling [13,15,16]. Once this regenerative cascade is successfully initiated, the tissue appears to undergo structural recovery autonomously [37,38,40].

Conversely, in conditions with a predominant central sensitization or nociplastic component (such as the myofascial pain syndrome evaluated by Yau et al. [43]), pain intensity tended to relapse—evidenced by a positive Δ in NRS scores—during the retention phase. This divergence suggests that while structural tissue repair can proceed autonomously, centrally mediated pain disorders may lack the capacity to maintain long-term neuromodulation without continuous peripheral stimulation [84,86,91]. In these nociplastic conditions, the persistence of pro-inflammatory cytokines (e.g., IL-1β, TNF-α, and IL-6) and the ongoing synthesis of prostaglandins (e.g., PGE_2_) likely continue to drive central sensitization pathways [4,13,84]. Although PEMF and EMTT provide effective initial analgesia by modulating adenosine receptors and reducing acute inflammatory markers, these effects may be transient if the underlying neuro-inflammatory state remains unaddressed by concurrent cognitive-behavioral or pharmacological interventions [28,29,92].

The present review found minimal reported adverse events across the included clinical studies. However, the safety of these high-energy protocols (up to 80–105 mT) is more robustly supported by contemporary in vitro experimental evidence. Recent mechanistic studies have demonstrated that exposing human tenocytes, osteoblasts, and mesenchymal stem cells to high-energy EMTT parameters does not compromise cellular viability [37,38,40]. Crucially, these high-intensity fields did not induce cytotoxicity, trigger apoptosis, or accelerate cellular senescence [40]. Instead, they exhibited senolytic-like effects and upregulated the expression of pro-regenerative genes (e.g., *RUNX2*, *COL1A1*, and *Scleraxis*) [37,40]. This translational evidence confirms that the high transfer rates required to reach deep targets do not cause thermal or mechanical damage to the cells, safely converting physical energy into biological repair [37].

Nevertheless, the absence of clinical adverse events in small trials must not be misinterpreted as definitive proof of absolute safety. High-energy electromagnetic fields have the potential to induce non-thermal cellular effects, including electroporation, which could theoretically cause undetected structural damage if dosages continue to escalate without standardized monitoring [93]. Future trials must implement standardized safety protocols to determine optimal biological thresholds.

To directly address the primary research question regarding the influence of EMTT and PEMF compared to sham, pharmacological, or conventional physical therapies, clinical outcomes must be interpreted through the hierarchy of available evidence. Across the 28 included studies, a clear dichotomy in treatment effect magnitude emerged when contrasting high-level randomized controlled trials (RCTs) with quasi-experimental designs and case reports.

In the lower tiers of evidence (quasi-experimental studies and case reports), electromagnetic therapies—particularly high-intensity EMTT combined with ESWT—consistently demonstrated substantial clinical trajectories. These observational studies frequently reported complete pain resolution, rapid radiographic bone consolidation, and full functional return to high-impact sports. While these uncontrolled observations substantiate the clinical feasibility and safety of high-energy interventions, they inherently risk overestimating treatment efficacy due to the absence of sham comparators and the confounding presence of highly active co-interventions.

The RCTs provided a more rigorous and nuanced answer to the research question. When specific high-intensity and high-cumulative-dose protocols were compared strictly against sham devices, they demonstrated true independent efficacy, consistently outperforming placebos in reducing pain and improving mechanical function. However, the most critical finding emerged when electromagnetic therapies were compared against, or added to, robust conventional physical therapies. In these scenarios, the RCTs frequently revealed that low-to-moderate intensity PEMF provided only marginal or statistically insignificant added benefits over standard rehabilitation. Active mechanical interventions, such as progressive resistance exercise, appeared to drive the bulk of the functional recovery, leaving a limited margin for the electromagnetic field to demonstrate independent superiority.

In summary, EMTT and PEMF positively influence pain reduction and functional recovery in adult patients with chronic musculoskeletal disorders, particularly when administered at high intensities and adequate cumulative doses. However, when subjected to the rigor of RCTs and compared directly against active conventional physical therapies, their independent superiority frequently diminishes. This suggests that rather than serving as standalone replacements for active physical rehabilitation, these electromagnetic modalities are most effectively utilized as advanced biological adjuncts to optimize the tissue environment for mechanical loading.

### 4.1. Implications for Research and Clinical Practice

The findings of this review highlight a critical methodological gap in the design and reporting of electromagnetic interventions. While this review utilized cumulative dose (mT·min) as a preliminary proxy to compare exposure across studies, we acknowledge that this metric is biophysically incomplete; it inherently ignores the crucial influence of frequency, specific waveforms, pulse counts, and the rate of magnetic field variation (dB/dt).

Ideally, evaluating the true electromagnetic stimulus requires calculating the actual transduction rate (e.g., utilizing formulas such as:|dB/dt|_max_ = 2πfB_0_ for continuous waves) where |dB/dt|_max_ represents the maximum rate of change in the magnetic field, f is the frequency, and B_0_ is the peak magnetic field intensity. However, performing these calculations retrospectively for the included studies was precluded by pervasive under-reporting. Most trials failed to specify coil geometry, exact pulse shapes, duty cycles, or rise times, necessitating a reliance on simplified metrics. Furthermore, an exhaustive re-evaluation revealed that the vast majority of studies failed to control or report the applicator-to-skin distance, applicator head type, and angle of radiation. As established by the inverse-square law, these positioning variables critically modify the effective magnetic flux density reaching the target tissue. This widespread lack of standardized reporting introduces a significant confounding variable regarding the true delivered dosimetry in current clinical evidence.

Therefore, this review serves as an urgent call to action for future clinical research. To transition from generalized electromagnetic applications to precision dosing, future trials must incorporate standardized reporting of all physical parameters: peak magnetic field intensity, frequency, waveform, and the calculated transduction rate (dB/dt). Furthermore, researchers must strictly define and report the applicator–patient interface—specifically controlling distance and angle of incidence—while adhering to international technical reporting guidelines for bioelectromagnetics to ensure the reproducibility of the delivered dose [94]. Only through transparent biophysical reporting and the integration of pain phenotyping tools will future meta-analyses be able to conclusively establish specific therapeutic thresholds.

### 4.2. Strengths and Limitations

However, these findings must be interpreted in light of several limitations. First, the marked clinical and methodological heterogeneity of the studies—ranging from diversity in models and biomarkers to the inclusion of case reports and the use of combination therapies (e.g., EMTT alongside ESWT)—precluded the performance of meta-analyses or formal statistical comparisons. This disparity in experimental designs, combined with the risk of bias identified in a portion of the primary evidence, makes it impossible to establish direct causality between physical parameters and clinical outcomes; therefore, the findings remain predominantly correlational.

Furthermore, it must be acknowledged as a methodological limitation that the original review protocol did not pre-specify quantitative analytical methods, such as meta-regression or sensitivity analysis, to evaluate the correlation between intensity and clinical outcomes. Consequently, the findings presented herein should be interpreted strictly as an exploratory and qualitative trend analysis rather than a statistical inference of causality. This lack of formal statistical dose–response analysis, necessitated by data heterogeneity, prevents the confirmation of whether physical variables independently affect outcomes.

Regarding the search strategy, the selection of information sources was focused on three primary databases: PubMed/MEDLINE, CENTRAL, and ScienceDirect. The decision to exclude additional databases such as Embase, Web of Science, CINAHL, and PEDro was based on the high degree of overlap these sources share with PubMed and CENTRAL within the field of physical agents and rehabilitation. Specifically, CENTRAL is recognized for its systematic indexing of randomized controlled trials from specialized databases like PEDro. Although an exhaustive manual search and direct contact with authors were implemented to mitigate potential bias, the omission of these specialized and technical databases represents a methodological limitation. This exclusion may have influenced the retrieval of highly specific studies from niche allied health journals or rapidly evolving technical fields.

A critical clinical limitation identified is the lack of data concerning the sustained effect of these interventions. Most included studies focus on immediate post-intervention outcomes, with limited or non-existent long-term follow-up. While PEMF and EMTT demonstrate promising acute efficacy, the paucity of robust evidence regarding effect retention at 6 or 12 months precludes confirming whether these therapies offer a definitive solution or require maintenance protocols. Consequently, future clinical trials must incorporate extended follow-up periods to establish long-term clinical relevance.

A significant technical limitation identified across the included literature is the lack of standardized reporting regarding positioning variables. Most studies limit their descriptions to device-specific parameters without specifying the applicator–patient interface. This omission introduces a substantial confounding variable in the assessment of cumulative dosimetry and hinders the reproducibility of the effective dose. Consequently, there is an urgent need for future clinical trials to adhere to technical reporting recommendations—such as those from the International Society for Electromagnetics in Surgery (ISES) or similar guidelines—to ensure rigorous dose reporting.

Moreover, although observed trends suggest a benefit associated with higher cumulative exposure, the lack of standardization in reporting transduction rates and intensity in the current literature reinforces the impossibility of confirming a definitive dose–response relationship. Additionally, as several of the biological mechanisms analyzed are derived from experimental and preclinical evidence, their extrapolation to the human clinical context must be approached with caution.

In addition to the above, the safety assessment of these interventions is subject to significant restrictions. Although the reviewed evidence suggests that the therapies were “well-tolerated within the analyzed experimental frameworks,” this observation should not be interpreted as definitive proof of absolute safety. Inconsistent reporting of adverse events and the absence of standardized pharmacovigilance protocols in the primary studies, coupled with small sample sizes, preclude the generalization of a definitive safety profile at this stage.

Finally, due to the high heterogeneity of the included designs and the inherent technical variability between EMTT and PEMF, the formal application of the GRADE (Grading of Recommendations Assessment, Development and Evaluation) system was deemed unfeasible for this study. Given the exploratory nature of this review and the prevalence of small sample sizes, the use of GRADE would have uniformly resulted in a “very low” certainty rating, offering no discriminatory value to the technical analysis. Instead, priority was given to a critical analysis focused on physical parameters and dosage trends to avoid underestimating technical findings that are fundamental for generating future hypotheses.

## 5. Conclusions

This systematic review highlights the potential of Pulsed Electromagnetic Fields (PEMF) and Extracorporeal Electromagnetic Transduction Therapy (EMTT) as adjunctive interventions in the treatment of musculoskeletal disorders. The integration of physical parameters suggests that clinical efficacy is closely linked to electromagnetic dosimetry, specifically field intensity and the transduction rate.

However, the robustness of these conclusions is constrained by methodological limitations inherent in the primary literature. The marked heterogeneity of study designs, coupled with the frequent use of combination therapies, precludes isolating the net effect of each modality and establishing a direct causal relationship. Since a formal quantitative analysis (such as meta-regression or sensitivity analysis) to evaluate the correlation between intensity and clinical outcomes was not pre-specified, the findings presented herein must be interpreted strictly as an exploratory and qualitative trend analysis rather than a statistical inference of independent causality. Consequently, these results serve as a foundation for hypothesis generation rather than a confirmation that physical variables affect outcomes in isolation.

Regarding safety, although the modalities were well-tolerated within the reviewed experimental frameworks, the lack of standardized adverse event reporting prevents the establishment of a definitive safety profile. This translational gap underscores the necessity for future research to prioritize randomized controlled trials with rigorous technical reporting and long-term follow-up. Only through the standardization of critical variables—including waveform, dB/dt, and systematic pharmacovigilance—can these qualitative trends be transformed into robust, reproducible, and statistically validated therapeutic recommendations.

## Figures and Tables

**Table 1:** Methodological design and clinical characteristics of the included studies.

Characteristic	Result
Included studies	28
Total participants	1049
Clinical trials	18
Quasi-experimental studies	2
Case reports	8
Clinical conditions assessed	27
Technologies assessed	EMTT and PEMF
Comparators	Sham, physiotherapy, therapeutic exercise, conventional treatment, and other physical therapies
Individual records extracted	361
Clinical domains analyzed	10

**Table 2:** Distribution of clinical outcomes categorized by therapeutic domain.

Clinical Domain	Records (n)	%
Function	151	41.8
Pain	96	26.6
Quality of life	33	9.1
Safety and feasibility	23	6.4
Imaging and tissue consolidation	19	5.3
Mental health	19	5.3
Electrophysiology	8	2.2
Inflammation and biomarkers	5	1.4
Clinical symptoms	3	0.8
Patient global perception	3	0.8
Other	1	0.3
Total	361	100

**Table 3:** Electromagnetic dosing parameters and qualitative pain outcomes of EMTT and PEMF interventions.

Study	Clinical Condition	Electromagnetic Prescription	Comparator/Co-Interventions	Main Qualitative Pain Outcome
**Randomized Controlled Trials–EMTT**				
Krath et al., 2017 [41]	Chronic non-specific low back pain	EMTT; 80 mT; 3 Hz; 20 min/session; 8 sessions	Conventional physiotherapy, superficial heat therapy, rescue analgesics.	EMTT combined with conventional care produced greater and more sustained pain reduction than conventional treatment alone.
Klüter et al., 2018 [42]	Non-calcific rotator cuff tendinopathy	EMTT; 80 mT; 3 Hz; 20 min/session; 8 sessions	Focused ESWT, scapular stabilization exercises, rescue paracetamol.	Combined EMTT and ESWT improved pain more than ESWT alone; however, the independent contribution of EMTT cannot be isolated because of the active co-intervention.
Yau et al., 2025 [43]	Chronic myofascial pain syndrome	EMTT; 80% maximum output; 3 Hz; 20 min/session; 8 sessions	Sham EMTT; no additional rehabilitation reported.	Pain improved following treatment, although no clinically meaningful superiority over sham was demonstrated.
**PEMF**				
Ammar, 2016 [54]	Chronic lateral epicondylopathy	PEMF; 25 Hz → 4.6 Hz; 30 min/session; 12 sessions	MENS plus wrist strengthening and stretching; exercise alone.	PEMF produced the greatest overall pain reduction among the three rehabilitation strategies.
Kamel et al., 2017 [55]	Postnatal carpal tunnel syndrome	PEMF; 8 mT; 50 Hz; 30 min/session; 12 sessions	Pulsed ultrasound; nerve- and tendon-gliding exercises.	PEMF achieved greater pain relief than pulsed ultrasound.
Lisi et al., 2019 [56]	Non-specific low back pain	Portable PEMF; home application for 12 weeks	Sham portable PEMF; usual medical care.	Both groups experienced clinically relevant pain improvement without significant between-group differences.
Elshiwi et al., 2019 [57]	Chronic non-specific low back pain	PEMF; 2 mT; 50 Hz; 20 min/session; 12 sessions	Sham PEMF; TENS, continuous ultrasound, lumbar strengthening and stretching.	Adjunctive PEMF did not provide clinically meaningful additional pain relief beyond conventional rehabilitation.
Karakaş and Gök, 2020 [58]	Chronic mechanical neck pain	PEMF; 2 mT; 10–100 Hz; 20 min/session; 15 sessions	Sham PEMF; hot pack, TENS and home exercise programme.	Both groups improved similarly, without superiority of PEMF over sham.
Nayback-Beebe et al., 2017 [67]	Chronic low back pain in military personnel	PEMF; 30 min/session; 12 sessions	Education, usual medication, stretching and strengthening exercises in both groups.	PEMF did not demonstrate significant superiority in pain reduction over standard care and exercise.
Elboim-Gabyzon and Nahhas, 2023 [61]	Knee osteoarthritis	PEMFT; 10 mT; 30 Hz; 15 min/session; 6 sessions	Low-level laser therapy; habitual activities maintained.	PEMFT produced greater short-term pain reduction than low-level laser therapy.
Ahmed et al., 2023 [62]	Lumbar myofascial pain syndrome	Low-intensity PEMF; 33 Hz; 15 min/session; 12 sessions	Conventional physiotherapy (plus a diathermy arm).	PEMF improved pain beyond conventional physiotherapy, although deep electrotherapy achieved the largest treatment effect.
Factor et al., 2023 [60]	Acute distal radius fracture	Portable PEMF; 0.05–0.5 mT; 10 Hz; continuous use (24 h/day)	Sham portable PEMF; cast immobilization followed by rehabilitation.	Continuous PEMF yielded rapid and sustained improvements in the PRWE pain subscale compared with sham.
Servodio Iammarrone et al., 2016 [66]	Patellofemoral pain syndrome	PEMF; 1.5 mT; 75 Hz; 240 min/day for 6 weeks	Home Exercise Program (HEP) including quadriceps, hip and gluteal strengthening.	PEMF combined with HEP produced greater long-term pain reduction than HEP alone, facilitating adherence to exercise.
Xu et al., 2024 [68]	Knee osteoarthritis	PEMF alone or combined with PRP	PRP alone; PEMF alone; PRP + PEMF.	The combined PRP + PEMF intervention demonstrated greater clinical improvement than either treatment alone; however, the specific contribution of PEMF cannot be isolated.
Yabroudi et al., 2024 [63]	Mild-to-moderate knee osteoarthritis	PEMF; 5 mT; 50 Hz; 30 min/session; 24 sessions	Progressive resistance exercise programme.	Addition of PEMF did not provide additional pain reduction beyond progressive resistance exercise.
Dündar et al., 2016 [64]	Bilateral knee osteoarthritis	PEMF; 0.1 mT; 50 Hz; 20 min/session; 20 sessions	Sham PEMF; heat, ultrasound, TENS and quadriceps isometric exercises.	Both groups experienced pain reduction without a clinically relevant advantage for PEMF.
Wuschech et al., 2015 [65]	Knee osteoarthritis	PEMF; 105 mT; 4–12 Hz; 5 min/session; 36 sessions	Sham MAGCELL^®^ device.	High-intensity PEMF produced greater pain reduction than placebo.
Hartard et al., 2023 [59]	Non-specific back pain	High-energy PEMF; 50 mT; 10 min/session; 3 sessions	Sham high-energy PEMF.	High-energy PEMF produced rapid and clinically relevant pain reduction compared with sham.
**Quasi-experimental Studies–EMTT**				
Gerdesmeyer et al., 2017 [70]	Midportion Achilles tendinopathy	EMTT; 80 mT; 3 Hz; 20 min/session; 8 sessions	Heel cushions.	EMTT demonstrated greater pain improvement than heel cushions alone; however, the non-randomized design limits causal inference.
Klüter et al., 2018 [69]	Chronic aseptic osteitis pubis	EMTT; 80 mT; 2.5 Hz; 15 min/session; 4 sessions	None; rescue analgesics as needed.	Rapid and sustained pain resolution was observed following treatment.
**Case Reports**				
Knobloch, 2021 [71]	Scaphoid non-union	Magnetolith^®^ EMTT: 8 Hz; energy level 8; 6000 impulses/session; 3 sessions + ESWT	Cast immobilization followed by strengthening.	Complete pain resolution accompanied fracture union.
Knobloch, 2025 [72]	Fifth metacarpal non-union	Magnetolith^®^ EMTT: 8 Hz; energy level 8; 6000 impulses/session; 3 sessions + ESWT	Orthotic immobilization.	Progressive pain resolution accompanied fracture consolidation.
Knobloch, 2022 [74]	Medial meniscal tear	Magnetolith^®^ EMTT: 8 Hz; energy level 8; 6000 impulses/session; 3 sessions + ESWT	Quadriceps strengthening and proprioceptive training.	Pain resolved with return to recreational running following combined therapy.
Knobloch, 2022 [75]	Humeral shaft non-union	Magnetolith^®^ EMTT: 8 Hz; energy level 8; 6000 impulses/session; 3 sessions + ESWT	Conservative immobilization.	Pain resolution paralleled progressive bone healing.
Knobloch et al., 2025 [72]	Hamulus ossis hamati non-union	Magnetolith^®^ EMTT: 8 Hz; energy level 8; 4000 impulses/session; 5 sessions + ESWT	Cast immobilization followed by strengthening.	Complete pain resolution and restoration of wrist function were reported following combined treatment.
Mohammadi et al., 2024 [78]	Femoral neck stress fracture	Magnetolith^®^ EMTT: 8 Hz; energy level 8; 6000 impulses/session; 3 sessions + HBOT	Hyperbaric oxygen therapy (HBOT).	Complete groin pain resolution enabled unrestricted return to long-distance running.
Gerdesmeyer et al., 2024 [76]	Clavicle fracture	Magnetolith^®^ EMTT: 8 Hz; energy level 8; 14,400 impulses/session; 5 sessions	Standard immobilization and progressive ROM physiotherapy.	Full pain resolution allowed return to professional triathlon performance.

**Abbreviations:** EMTT, extracorporeal magnetotransduction therapy; ESWT, extracorporeal shock wave therapy; HBOT, hyperbaric oxygen therapy; HEP, home exercise program; MENS, microcurrent electrical nerve stimulation; PEMF, pulsed electromagnetic field; PEMFT, pulsed electromagnetic field therapy; PRP, platelet-rich plasma; PRWE, Patient-Rated Wrist Evaluation; ROM, Range of Motion; TENS, transcutaneous electrical nerve stimulation; Hz, hertz; mT, millitesla; min, minutes.

**Table 4:** Clinical functional outcomes associated with EMTT and PEMF interventions.

Study	Clinical Condition	Detailed Rehabilitation Programme/Co-Interventions	Functional Assessment Tools (Categorized)	Main Qualitative Functional Outcome
**Randomized Controlled Trials—EMTT**				
Krath et al., 2017 [41]	Chronic non-specific low back pain	Conventional physiotherapy, superficial heat therapy, rescue analgesia (ibuprofen/metamizole); no structured exercise programme.	**General Disability:** Oswestry Disability Index (ODI)	EMTT improved disability and functional performance beyond conventional physiotherapy.
Klüter et al., 2018 [42]	Non-calcific rotator cuff tendinopathy	Three sessions of focused ESWT and identical scapular stabilization exercises in both groups.	**Joint-Specific:** Constant-Murley Score	Combined EMTT and ESWT produced greater improvement in shoulder function than ESWT alone.
Yau et al., 2025 [43]	Chronic myofascial pain syndrome	No additional rehabilitation programme reported; applied directly over the most painful region.	**General Disability:** Brief Pain Inventory (BPI)—Interference Scale	Functional performance improved, although no clinically meaningful superiority over sham was demonstrated.
**Randomized Controlled Trials—PEMF**				
Ammar, 2016 [54]	Chronic lateral epicondylopathy	Progressive wrist extensor strengthening, static stretching, and home exercises in all groups.	**Joint-Specific:** DASH; **Objective Performance:** Hand dynamometer	PEMF produced the greatest improvement in upper-limb function among the three rehabilitation strategies.
Kamel et al., 2017 [55]	Postnatal carpal tunnel syndrome	Median nerve- and tendon-gliding exercises in both groups.	**Joint-Specific:** CTSQ Functional Status Scale; **Objective Performance:** Hydraulic dynamometer	PEMF achieved greater improvement in hand function and grip strength than pulsed ultrasound.
Lisi et al., 2019 [56]	Non-specific low back pain	Usual medical care at clinical discretion; no structured rehabilitation programme restricted.	**General Disability:** Oswestry Disability Index (ODI)	Functional disability improved similarly in both groups, with no significant between-group differences.
Elshiwi et al., 2019 [57]	Chronic non-specific low back pain	TENS (100 Hz, 15 min, 3×/week), continuous ultrasound (1 MHz, 1.5 W/cm^2^, 5 min), and individualized lumbar strengthening and stretching.	**General Disability:** ODI; **Objective Performance:** Modified Schober test, Finger-to-floor distance	Both groups demonstrated functional improvement; adjunctive PEMF did not provide additional benefit.
Karakaş and Gök, 2020 [58]	Chronic mechanical neck pain	Hot pack, TENS, and a home exercise programme emphasizing cervical mobility and strengthening; rescue paracetamol permitted.	**Joint-Specific:** Neck Pain Disability Scale; **General Disability:** Patient Global Assessment (PGA)	Cervical disability improved in both groups without superiority of PEMF over sham.
Elboim-Gabyzon and Nahhas, 2023 [61]	Knee osteoarthritis (KL 2–3)	Maintenance of habitual activities; no additional rehabilitation intervention.	**Joint-Specific:** WOMAC (total, stiffness, physical function); **Objective Performance:** TUG, 10-Meter Walk Test (10MWT)	PEMF demonstrated greater improvement in physical function and mobility than low-level laser therapy.
Ahmed et al., 2023 [62]	Lumbar myofascial pain syndrome	Therapeutic ultrasound, stretching (hamstrings, gastrocnemius, lumbar muscles), and progressive strengthening exercises.	**General Disability:** Oswestry Disability Index (ODI)	PEMF improved functional disability beyond conventional physiotherapy.
Factor et al., 2023 [60]	Acute distal radius fracture	Cast immobilization; progressive rehabilitation programme initiated strictly after cast removal.	**Objective Performance:** Jamar dynamometer, goniometry (ROM)	Continuous PEMF accelerated functional recovery, significantly improving grip strength and range of motion.
Servodio Iammarrone et al., 2016 [66]	Patellofemoral pain syndrome	Home exercise programme consisting of quadriceps, hip adductor, and gluteal strengthening, balance training, and lower-limb stretching.	**Joint-Specific:** VISA-P, Feller Patella Score	PEMF combined with a home exercise programme produced greater long-term functional improvement than exercise alone.
Xu et al., 2024 [68]	Early-stage knee osteoarthritis	Intra-articular injection of leukocyte-poor platelet-rich plasma (LP-PRP) according to group allocation.	**Joint-Specific:** WOMAC Total, Lequesne Index; **Objective Performance:** Knee ROM	Combined PRP and PEMF achieved the greatest functional improvement.
Yabroudi et al., 2024 [63]	Mild-to-moderate knee osteoarthritis	Supervised progressive resistance training (PRE) targeting the lower extremities.	**Joint-Specific:** KOOS (total, symptoms, ADL, sport/recreation); **Objective Performance:** 4 m walk, Five-Times Sit-to-Stand Test	Progressive resistance exercise substantially improved function; adjunctive PEMF did not provide additional benefit.
Dündar et al., 2016 [64]	Bilateral knee osteoarthritis	Heat therapy, therapeutic ultrasound, TENS, and quadriceps isometric strengthening exercises.	**Joint-Specific:** WOMAC	Functional improvement occurred in both groups without a clinically relevant advantage for PEMF.
Wuschech et al., 2015 [65]	Knee osteoarthritis	No structured rehabilitation programme or co-intervention reported.	**Joint-Specific:** WOMAC (total, physical function)	High-intensity PEMF produced greater improvements in physical function than placebo.
**Quasi-experimental Studies—EMTT**				
Gerdesmeyer et al., 2017 [70]	Midportion Achilles tendinopathy	Bilateral 1 cm heel cushions; no structured exercise programme reported.	**Joint-Specific:** Roles-Maudsley Score	EMTT improved Achilles tendon-related function more than heel cushions.
Klüter et al., 2018 [69]	Chronic aseptic osteitis pubis	Rescue analgesia permitted (ibuprofen/metamizole); no additional rehabilitation programme.	**Objective Performance:** Return to sports participation	Progressive return to unrestricted sports participation and normal function was reported.
**Case Reports—Combined Interventions**				
Knobloch Series (2020–2025) [72,73,74,75,77]	Various non-unions and meniscal tears	Splint/cast immobilization followed by targeted progressive strengthening and proprioceptive training.	**Joint-Specific:** DASH; **Objective Performance:** Clinical evaluation, Running mileage	Full joint function and return to sports were consistently restored following fracture union.
Mohammadi et al., 2024 [78]	Femoral neck stress fracture	Hyperbaric oxygen therapy (HBOT), core strengthening, hip mobility, and gradual return to running.	**Objective Performance:** Manual Muscle Testing (MMT), Return to sports	Functional recovery enabled unrestricted return to long-distance running.
Gerdesmeyer et al., 2024 [76]	Clavicle fracture	Standard immobilization and progressive ROM physiotherapy.	**Objective Performance:** Return to sports	Full functional recovery allowed return to professional triathlon performance.

**Abbreviations**: EMTT, extracorporeal magnetotransduction therapy; ESWT, extracorporeal shock wave therapy; HBOT, hyperbaric oxygen therapy; PEMF, pulsed electromagnetic field; PRP, platelet-rich plasma; TENS, transcutaneous electrical nerve stimulation; Hz, hertz; mT, millitesla; min, minutes.

**Table 5:** Summary of Exercise Co-interventions and Active Control Protocols Across Included Trials.

Control Category	Primary Biomechanical/Physiological Objective	Representative Studies	Expected Influence on Baseline Recovery
**Progressive Resistance Exercise (PRE)**	Promote tendon, muscle, and periarticular tissue remodeling through progressive mechanical loading, stimulating collagen synthesis, tenocyte activity, muscle hypertrophy, and load tolerance.	Yabroudi et al. [63]; Ammar [54]; Servodio Iammarrone et al. [66]; Elshiwi et al. [57]	Provides a strong biological and mechanical stimulus for tissue adaptation, resulting in a high baseline potential for pain reduction and functional recovery, thereby reducing the detectable incremental effect of adjunctive electromagnetic therapy.
**Isometric Exercise**	Produce immediate analgesia through neuromuscular activation, cortical inhibitory mechanisms, and motor unit recruitment while minimizing joint movement and tissue strain.	Dündar et al. [64]	Primarily improves short-term pain modulation and muscle activation, with limited capacity to induce structural tissue remodeling compared with progressive resistance exercise.
**Motor Control and Stabilization Exercise**	Restore optimal movement patterns, joint kinematics, proprioception, and neuromuscular coordination by improving segmental stability and motor control.	Klüter et al. [42]; Kamel et al. [55]	Corrects dysfunctional movement strategies and enhances joint stability, acting synergistically with tissue-healing interventions while reducing recurrent mechanical overload.
**Passive Physical Modalities/Usual Care**	Provide symptomatic relief through physical agents (e.g., heat therapy, ultrasound, TENS) without delivering sufficient mechanical loading to stimulate structural tissue adaptation.	Krath et al. [41]; Lisi et al. [56]; Wuschech et al. [65]	Produces predominantly palliative effects, leaving biological tissue remodeling dependent on spontaneous healing or adjunctive regenerative interventions such as EMTT or PEMF.

**Table 6:** Summary of Health-Related Quality of Life (HRQoL) Outcomes Following EMTT and PEMF Interventions.

Study	Clinical Condition	HRQoL Instrument	Main Qualitative Outcome
**Randomized Controlled Trials—EMTT**			
Yau et al., 2025 [43]	Chronic myofascial pain syndrome	SF-36	Within-group improvements were observed in several health-related quality-of-life domains; however, no clinically meaningful superiority over sham treatment was demonstrated.
**PEMF**			
Factor et al., 2023 [60]	Acute distal radius fracture	SF-12	Continuous PEMF significantly improved the physical component of health-related quality of life compared with sham, whereas no between-group differences were observed for the mental component.
Karakaş and Gök, 2020 [58]	Chronic mechanical neck pain	SF-36	Both groups demonstrated improvements in health-related quality of life following treatment; adjunctive PEMF did not provide additional benefit over sham.
Nayback-Beebe et al., 2017 [67]	Chronic low back pain	SF-36	Physical health-related quality of life improved after PEMF treatment, whereas no clinically meaningful improvement was observed in pain or disability. Anxiety scores increased during follow-up.
Yabroudi et al., 2024 [63]	Knee osteoarthritis	KOOS–QoL	Knee-related quality of life improved following rehabilitation; however, adding PEMF to progressive resistance exercise did not provide additional benefit.

**Abbreviations:** EMTT, extracorporeal magnetotransduction therapy; HRQoL, health-related quality of life; KOOS–QoL, Knee Injury and Osteoarthritis Outcome Score–Quality of Life subscale; PEMF, pulsed electromagnetic field; SF-12, 12-Item Short Form Health Survey; SF-36, 36-Item Short Form Health Survey. **Notes:** Only studies reporting validated health-related quality-of-life (HRQoL) instruments are included in this table. Disease-specific functional scales (e.g., WOMAC, ODI, PRTEE, Kujala Score, Constant-Murley Score, VISA-A, KOOS-ADL) are summarized in Table 4. Qualitative outcomes reflect the findings reported in the original studies, with an emphasis on clinically relevant between-group differences where applicable.

**Table 7:** Quantitative Analysis of the Retention Effect: Mean Changes (Δ) from Post-Intervention to Final Follow-up.

Study	Clinical Condition	Score at End of Therapy (Initial Post-Intervention)	Score at Last Follow-Up	Retention Delta (Δ) *	Clinical Interpretation
**RCTs—Structural/Degenerative**					
Krath et al., 2017 (EMTT) [41]	Chronic low back pain	ODI: 25.6 (at 12 weeks)	ODI: 20.8 (at 18 weeks)	Δ −4.8	Continued functional improvement
Xu et al., 2024 (PEMF) [68]	Early-stage knee OA	WOMAC: 32.7 (at 4 weeks)	WOMAC: 20.9 (at 12 weeks)	Δ −11.8	Continued functional improvement
Servodio Iammarrone et al., 2016 (PEMF) [66]	Patellofemoral pain	Feller Score: 24.0 (at 2 months)	Feller Score: 28.5 (at 12 months)	Δ +4.5	Continued functional improvement
Factor et al., 2023 (PEMF) [60]	Distal radius fracture	Grip Strength: 7.5 kg (at 6 weeks)	Grip Strength: 14.2 kg (at 12 weeks)	Δ +6.7 kg	Massive continued strength gain
Yabroudi et al., 2024 (PEMF + PRE) [63]	Mild-to-moderate knee OA	KOOS ADL: 82.2 (at 8 weeks)	KOOS ADL: 73.3 (at 6 months)	Δ −8.9	Slight functional decline over time
**RCTs—Nociplastic/Myofascial**					
Yau et al., 2025 (EMTT) [43]	Myofascial pain syndrome	NRS-11: 4.3 (at 4 weeks)	NRS-11: 5.6 (at 6 months)	Δ +1.3	Pain recurrence post-treatment
**Quasi-experimental Studies**					
Klüter et al., 2018 (EMTT) [69]	Aseptic osteitis pubis	VAS (0–10): 0.5 (Immediate post)	VAS (0–10): 0.0 (at 3 months)	Δ −0.5	Complete pain resolution maintained
**Case Reports**					
Knobloch Series, 2020–2025 [71,72,73,74]	Non-unions (various)	Clinical union achieved	Follow-ups at 6 months	N/A	100% maintenance of radiographic union and pain resolution

* The retention delta (Δ) calculated the difference between post-treatment and final follow-up scores to evaluate clinical stability after the intervention: The mathematical direction of clinical improvement varies by instrument. A negative delta (Δ) denotes a continued reduction in symptoms (e.g., further decreases in ODI, WOMAC, NRS, or VAS scores). Conversely, a positive delta (Δ) indicates sustained gains in performance or function (e.g., further increases in Feller Score or grip strength).

**Table 8:** Summary of Adverse Events and Safety Profiles Across Included.

Study (Author, Year)	Clinical Condition	Reported Adverse Event(s)	Severity	Related to EMTT/PEMF	Clinical Outcome/Interpretation
Gerdesmeyer et al., 2017 [70]	Midportion Achilles tendinopathy	Minor transient skin redness at the treatment site.	Mild	Yes	Self-limiting local reaction that resolved spontaneously without treatment discontinuation.
Krath et al., 2017 [41]	Chronic non-specific low back pain	Transient skin erythema that resolved within approximately 1 h after treatment.	Mild	Yes	Temporary local reaction with spontaneous resolution; no clinically significant complications were reported.
Klüter et al., 2018 [42]	Non-calcific rotator cuff tendinopathy	Petechiae, small cutaneous hematoma, and transient erythema following treatment.	Mild	Yes	Local superficial tissue reactions that resolved without sequelae and did not require treatment interruption.
Lisi et al., 2019 [56]	Non-specific low back pain	One sports-related injury and one episode of intrauterine device (IUD)-related spotting. Both were judged unrelated to the study intervention. Adverse events were rare and mild.	Mild	No	No treatment-related adverse events were identified. The portable PEMF device demonstrated a favorable safety profile.
Xu et al., 2024 [68]	Early-stage knee osteoarthritis	Mild knee swelling, transient fever, and temporary exacerbation of osteoarthritis pain following treatment.	Mild	Yes (mainly associated with combined PRP/PEMF treatment)	All events resolved with conservative management and no serious adverse events were reported.
Yau et al., 2025 [43]	Chronic myofascial pain syndrome	Temporary increase in pain intensity after treatment.	Mild	Yes	Short-lived exacerbation that resolved spontaneously without discontinuation of therapy.

## Data Availability

The original contributions presented in this study are included in the article. Further inquiries can be directed to the corresponding author.

## Supplementary Materials

The following supporting information can be downloaded at: https://www.mdpi.com/article/10.3390/biomedicines14081731/s1.

## Abbreviations

The following abbreviations are used in this manuscript:

CRMRF	capacitive-resistive monopolar radiofrequency
CSI	Central Sensitization Inventory
CTSQ	Carpal Tunnel Syndrome Questionnaire
DASH	Disabilities of the Arm, Shoulder and Hand
EMTT	extracorporeal magnetotransduction therapy
ESWT	extracorporeal shock wave therapy
EVA	Visual Analogue Scale
HE-PEMF	high-energy pulsed electromagnetic field
JBI	Joanna Briggs Institute
LPEMF	low-intensity pulsed electromagnetic field
NRS	Numeric Rating Scale
ODI	Oswestry Disability Index
PEMF	pulsed electromagnetic fields
PRISMA	Preferred Reporting Items for Systematic Reviews and Meta-Analyses
PRP	platelet-rich plasma
QST	Quantitative Sensory Testing
SF-12	Short Form-12 Health Survey
ROM	range of motion
SF-36	Short Form-36 Health Survey
TUG	Timed Up and Go
VAS	Visual Analogue Scale
WOMAC	Western Ontario and McMaster Universities Osteoarthritis Index
