# Supplementary material for: Contemporary Advances (2015–2026) in Extracorporeal Electromagnetic Transduction Therapy and Pulsed Electromagnetic Fields for Musculoskeletal Disorders: A Systematic Review of Dosimetry and Clinical Response
Source: Biomedicines. 2026 Jul 31;14(8):1731. doi: 10.3390/biomedicines14081731 (PMC13509872; doi:10.3390/biomedicines14081731)
Supplement: Supplementary file 1 [file biomedicines-14-01731-s001.zip › biomedicines-4447704-supplementary.pdf]

Supplementary File S1: Detailed Search Strategy and Eligibility Criteria

1. Research Question and PICO Framework The systematic review was designed to answer the following research question: *What is the effectiveness of extracorporeal magnetotransduction therapy (EMTT) and conventional pulsed electromagnetic fields (PEMF)—administered at specific intensities (mT) and frequencies (Hz)—compared to sham, pharmacological, or conventional physical therapies in reducing pain and improving function in adult patients with chronic musculoskeletal disorders?*

- **P (Population):** Adult patients (>18 years of age) with chronic musculoskeletal injuries, including stable upper/lower extremity fractures, non-unions, lateral/medial epicondylopathy, rotator cuff tears, knee osteoarthritis, and non-specific chronic low back pain.
- **I (Intervention):** Extracorporeal magnetotransduction therapy (EMTT) or Pulsed Electromagnetic Fields (PEMF).
- **C (Comparator):** Conventional physical therapy, pharmacological treatment (analgesics, NSAIDs), therapeutic exercise, or sham/placebo interventions.
- **O (Outcomes):** Reduction in pain intensity and improvement in functional recovery.

2. Eligibility Criteria

Inclusion Criteria:

- **Clinical Conditions:** Stable fractures of the extremities (radiographically confirmed); non-unions/delayed unions (radiographically confirmed); lateral or medial epicondylopathy (clinically and radiographically confirmed); partial or complete rotator cuff tears (MRI confirmed); knee osteoarthritis (Kellgren-Lawrence grades 2 or 3, radiographically confirmed); chronic non-specific low back pain (clinically confirmed).
- **Population:** Adults aged >18 years (inclusion was determined based on the group mean age reported in the study, allowing for the preservation of external validity in highly prevalent conditions like osteoarthritis).
- **Interventions:** Protocols explicitly detailing the use of EMTT or PEMF parameters.
- **Comparators:** Sham procedures, conventional physiotherapy, therapeutic exercise, or pharmacological interventions.
- **Study Design:** Randomized controlled trials (RCTs), controlled clinical trials, quasi-experimental studies, and case reports.

- **Language:** Published in English or Spanish.
- **Timeframe:** Published between January 1, 2015, and the date of the search (May 31, 2026).

Exclusion Criteria:

- Patients with significant systemic comorbidities that could alter biological tissue repair (e.g., active rheumatoid arthritis, severe neurological diseases, uncontrolled diabetes mellitus).
- Concurrent distinct injuries that could significantly affect functional outcomes.
- Studies with high attrition rates or lost to follow-up without proper data imputation.
- In vitro studies, animal models, narrative reviews, editorials, and conference abstracts.

3. Database-Specific Search Strings

The search was conducted across three major databases: PubMed/MEDLINE, Cochrane Central Register of Controlled Trials (CENTRAL), and ScienceDirect. The exact search strings and applied filters are detailed below:

A. PubMed / MEDLINE

- **Search Date Range:** January 1, 2015 – May 2026
- **Search String:** (("Electromagnetic transduction therapy"[Title/Abstract] OR "EMTT"[Title/Abstract] OR "extracorporeal magnetotransduction therapy"[Title/Abstract] OR "physiomagneto"[Title/Abstract] OR "magnetolith"[Title/Abstract] OR "PEMF"[Title/Abstract] OR "Pulsed Electromagnetic Fields"[Title/Abstract])) AND (("randomized controlled trial"[Title/Abstract] OR "RCT"[Title/Abstract] OR "controlled clinical trial"[Title/Abstract] OR "quasi-experimental study"[Title/Abstract] OR "case report"[Title/Abstract]))
- **Filters Applied:**
    - Publication Date: 2015/01/01 to 2026/05/31
    - Article Types: Case Reports, Clinical Study, Clinical Trial, Clinical Trial Phase I-IV, Controlled Clinical Trial, Multicenter Study, Pragmatic Clinical Trial, Randomized Controlled Trial.

- Species: Humans
- Ages: Adult (19+ years), Middle Aged (45-64 years)
- Languages: English, Spanish

B. Cochrane Library (CENTRAL)

- **Search Date Range:** January 2015 – 2026
- **Search String (Searched in Title, Abstract, Keyword):** (("Electromagnetic transduction therapy" OR "EMTT" OR "extracorporeal magnetotransduction therapy" OR "physiomagneto" OR "magnetolith" OR "PEMF" OR "Pulsed Electromagnetic Fields") AND ("randomized controlled trial" OR "RCT" OR "controlled clinical trial" OR "quasi-experimental study" OR "case report"))
- **Filters Applied:**
    - Publication Year: 2015 to 2026
    - Database: Cochrane Central Register of Controlled Trials

C. ScienceDirect

- **Search Date Range:** 2015 – 2026
- **Search String:** ("Electromagnetic transduction therapy" OR "EMTT" OR "extracorporeal magnetotransduction therapy" OR "magnetolith" OR "PEMF" OR "Pulsed Electromagnetic Fields")
- **Filters Applied:**
    - Years: 2015, 2016, 2017, 2018, 2019, 2020, 2021, 2022, 2023, 2024, 2025, 2026
    - Article Type: Research Articles (FLA)
    - Language: English, Spanish
